# Deciphering microvascular changes after myocardial infarction through 3D fully automated image analysis

**DOI:** 10.1038/s41598-018-19758-4

**Published:** 2018-01-30

**Authors:** Polyxeni Gkontra, Kerri-Ann Norton, Magdalena M. Żak, Cristina Clemente, Jaume Agüero, Borja Ibáñez, Andrés Santos, Aleksander S. Popel, Alicia G. Arroyo

**Affiliations:** 1 Centro Nacional de Investigaciones Cardiovasculares Carlos III (CNIC), Madrid, 28029 Spain; 2 Department of Biomedical Engineering, School of Medicine, Johns Hopkins University, Baltimore, MD 21205 USA; 3 Centro de Investigación Biomédica en Red de Enfermedades CardioVasculares (CIBERCV), Madrid, Spain; 4 IIS-Fundación Jiménez Díaz, Madrid, Spain; 5 Biomedical Image Technologies (BIT), ETSI Telecomunicación, Universidad Politécnica de Madrid, Madrid, 28040 Spain; 6 Centro de Investigación Biomédica en Red de Bioingeniería, Biomateriales y Nanomedicina (CIBERBBN), Madrid, Spain; 7 Present Address: Division of Science, Mathematics, and Computing, Bard College, Annandale-on-Hudson, NY, 12504 USA

## Abstract

The microvasculature continuously adapts in response to pathophysiological conditions to meet tissue demands. Quantitative assessment of the dynamic changes in the coronary microvasculature is therefore crucial in enhancing our knowledge regarding the impact of cardiovascular diseases in tissue perfusion and in developing efficient angiotherapies. Using confocal microscopy and thick tissue sections, we developed a 3D fully automated pipeline that allows to precisely reconstruct the microvasculature and to extract parameters that quantify all its major features, its relation to smooth muscle actin positive cells and capillary diffusion regions. The novel pipeline was applied in the analysis of the coronary microvasculature from healthy tissue and tissue at various stages after myocardial infarction (MI) in the pig model, whose coronary vasculature closely resembles that of human tissue. We unravelled alterations in the microvasculature, particularly structural changes and angioadaptation in the aftermath of MI. In addition, we evaluated the extracted knowledge’s potential for the prediction of pathophysiological conditions in tissue, using different classification schemes. The high accuracy achieved in this respect, demonstrates the ability of our approach not only to quantify and identify pathology-related changes of microvascular beds, but also to predict complex and dynamic microvascular patterns.

## Introduction

Cardiovascular diseases (CVDs) are the leading cause of deaths worldwide, despite significant progress having been made in their prognosis, treatment and medical management^1^. The socio-economic burden associated with CVDs, including myocardial infarction (MI) and its complications such as heart failure^2^, has led an important amount of research to focus on unravelling its causes and developing therapeutic approaches^3^. In recent decades, dysfunction of the coronary microvasculature has emerged as an additional complication of myocardial ischaemia, and, therefore, as a potential prognostic biomarker and therapeutic target^4,5^.

The microvasculature represents the anatomy of microcirculation and comprises the smallest blood vessels of the tissues; capillaries, arterioles and venules. Its architecture is a major determinant of blood flow, oxygen transport, wall shear stress and distribution of pressure in microvessels^6,7^. Nonetheless, its *in vivo* visualization in humans remains a bottleneck. For this reason, *ex vivo* imaging and different animals models have been used^8–13^. Among them, the pig animal model has attracted considerable attention due to the similarity of its coronary network to that of humans. Pioneering work by Kassab^13^ in this area has provided a wealth of data regarding diameters, length and topology of the porcine microvascular network, through the use of coronary corrosion casts. These data provided significant insight into porcine microvascular structure and enabled the subsequent modelling of hemodynamics at basal conditions^14,15^.

However, the microvasculature is not a static system, but rather a dynamic one and it continuously adapts in order to meet the tissue demands in response to physiological and pathophysiological conditions, such as MI and other CVDs as diabetes or hypertension. Thanks to its dynamic nature, the microvasculature is capable of adding new vessels by sprouting^16^ or intussusceptive angiogenesis^17^, altering the structure of existing vessels (remodelling^18^) and pruning of abundant vessels (regression^19^). These processes of vascular patterning, termed angioadaptation^20^, affect the architecture of the microvasculature. Despite significant attempts to develop approaches that would promote therapeutic remodelling or revascularization^21^, no drug has so been approved for clinical use for MI^22,23^. There is, therefore, a growing quest to develop a better understanding of the microvascular dynamic changes in MI and how to control them. Towards this aim, quantitative data on the anatomy of coronary microcirculation in pathology and not only under basal conditions are essential^24^. These data are the key challenge for understanding structure-function relation through modelling approaches, and thus, developing and evaluating more efficient therapeutic approaches. In addition, studies are expected to recognize the inherently three-dimensional (3D) and complex structure of the microvasculature.

Obtaining such data is nowadays more feasible than ever thanks to revolutionary advancements in confocal microscopy that have allowed the acquisition of data in 3D with sub-micrometer resolution, at increasing imaging depths^25^. Furthermore, confocal microscopy, in combination with fluorescent dyes, allows the simultaneous study of the microvasculature in relation to other key players in tissue healing after MI. However, today’s imaging systems produce a vast amount of data whose complexity preclude traditional manual and supervised analysis methods^26^. A work-around adapted by the scientific community during several years has been the assessment of the microvasculature from 2D slices or maximum intensity projections produced from 3D volumes. However, it is increasingly apparent that this approach might lead to errors, misinterpretations and loss of important information of biological significance. Therefore, the quest for 3D automated bioimage analysis tools has steadily been increasing^27,28^.

In this work, we aim to enrich the knowledge about cardiac microcirculation pathophysiology with quantitative data spanning different stages following MI (1, 3, and 7 days), as well as from basal conditions, in an unbiased manner. We use thick slices of porcine cardiac tissue, label them for cell nuclei, endothelial cell junctions and smooth muscle actin positive (SMA^+^) cells and image them by using confocal microscopy. We design a 3D fully automatic bioimage analysis pipeline that firstly allows us to reconstruct the complete microvasculature from stained endothelial junctions and SMA^+^ cells. We subsequently extract essential biological information about all major characteristics of the microvasculature, its relation with SMA^+^ and capillary diffusion regions, which was not possible in the past when using a single software. We show that using confocal microscopy and our pipeline we are able to unravel fine alterations of the cardiac microvasculature in the aftermath of MI. The fully automatic nature of the approach ensures reproducibility, reliable significance and objectiveness of the findings. Furthermore, we evaluate the potential of the extracted knowledge to be used for predicting the pathophysiological condition of unseen tissue and we achieve high accuracy (higher than 80%) in the task of distinguishing infarcted from basal tissue and in recognizing the stage of the infarcted tissue. Lastly, we also investigate a later time point post MI (45 days) to understand whether early microvascular alterations persist and/or change during cardiac remodelling. Overall, the study allows a deeper understanding of microvascular alterations in MI and marks a step forward towards modelling of microcirculation at different stages after pathology, while it provides an unbiased means in the evaluation of the outcome of potential treatments.

## Results

### Endothelial cell junction-based 3D reconstruction of the microvasculature from cardiac tissue

We use thick slices of cardiac tissue (~100 *μ*m) stained with fluorescent markers for cell nuclei (Hoechst), endothelial junctions (anti-VE-Cadherin) and SMA^+^ cells (anti-α-SMA) and image them by means of confocal microscopy (see Methods). In the case of subjects that had suffered MI, tissues from both infarcted and non-infarcted (remote) areas are used. Prior to proceeding with the core of the analysis pipeline, non-local means filtering (NLMF)^29^ was applied with the aim of enhancing the quality of the images while preserving image information.

The first step towards quantifying the microvasculature and its infarction-related dynamic changes is to automatically segment the labelled structures of the VE-Cadherin, SMA and of the Hoechst channels. Towards this aim, the multi-scale multi-level thresholding algorithm (MMT)^30^ is applied to every channel of the 3D confocal image. In the case of the VE-Cadherin channel, the segmentation is further improved by excluding possible artefacts, i.e. objects that are smaller than 100 voxels and that do not have a nucleus.

Following the segmentation of structures labelled with VE-Cadherin, major parts of the microvasculature are reconstructed. However, VE-Cadherin is a marker of endothelial junctions and not of the vessel lumen or of the complete endothelial cell (see Methods). As a result, there are gaps in the reconstructed blood vessels. The size of these gaps is not homogeneous but rather depends on the type of microvessels. Smaller gaps are observed in capillaries and larger ones in larger microvessels, i.e. arterioles and venules. A filling method was developed to deal with this limitation. The method is applied on top of the VE-Cadherin segmentation with the aim of reconstructing microvascular parts which had not been labelled with VE-Cadherin.

**Figure 1:**
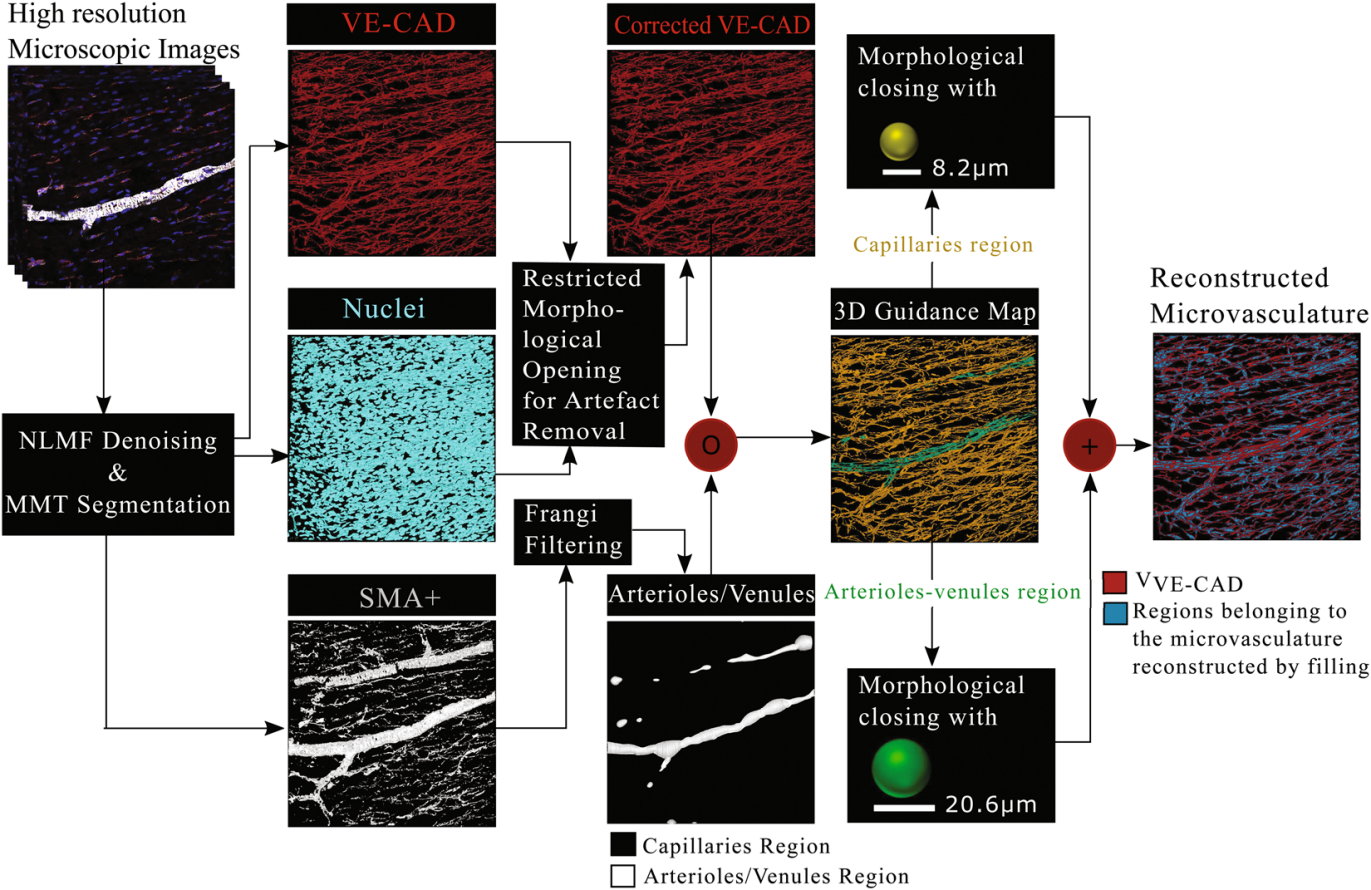
Overview of the proposed approach for the reconstruction of the complete microvasculature from thick sections of pig heart tissue labelled for VE-Cadherin (endothelial junctions).

An overview of the filling algorithm is provided in Fig. 1. The method is based on two types of information; information regarding the spatial relation of the microvessels with SMA^+^ cells and information about the physiology of the different types of microvessels that form the microvascular network. In particular, taking advantage of the knowledge about the existence of a SMA^+^ layer that surrounds arterioles and venules^31^, Frangi-based filtering^32^ is applied to the SMA^+^ segmentation to extract tubular elements whose diameter is in the range of that of arterioles and venules (i.e. larger than the diameter of 7.3 *μm* of the larger capillary connected directly to an arteriole^13^). This results in a 3D binary image on which regions that might belong to arterioles/venules are on voxels (voxels of value one) and regions belonging to capillaries are off voxels (voxels with value equal to zero). The 3D image is subsequently used to distinguish between microvessels formed by the VE-Cadherin junctions that are capillaries and those that are possible arterioles or venules. For this purpose, element-wise multiplication of the VE-Cadherin segmentation and the 3D image with arterioles and capillaries is performed. The procedure results in the creation of a 3D Guidance Map (Supplementary Fig. S1) containing information about the type of microvessels and therefore of the size of gaps that should be filled.

Morphological closing is performed using structural elements of various sizes based on the vessel type, as defined by the 3D Guidance Map. More precisely, in order to fill gaps in regions of capillaries, a ball structural element is used whose size is equal to the diameter of the largest capillary (8.2 *μm*) found in the pig animal model^13^. Similarly, in order to fill gaps in the case of arterioles/venules a larger ball structural element is used whose diameter is equal to the largest reported arteriole/venule of order 2 (20.6 *μm*). The combination of filled capillaries and arterioles/venules produced by means of the aforementioned work-flow results in the reconstruction of the complete microvasculature, including regions that might not have been labelled with VE-Cadherin, but nonetheless belong to the microvasculature. Supplementary Fig. S2 allows appreciation of the improved identification of the microvascular network by using the proposed filling approach.

### Myocardial infarction decreases 3D complexity, integrity, and connectivity of the coronary microvasculature while it leads to altered capacity and heterogeneity of blood flow

**Figure 2:**
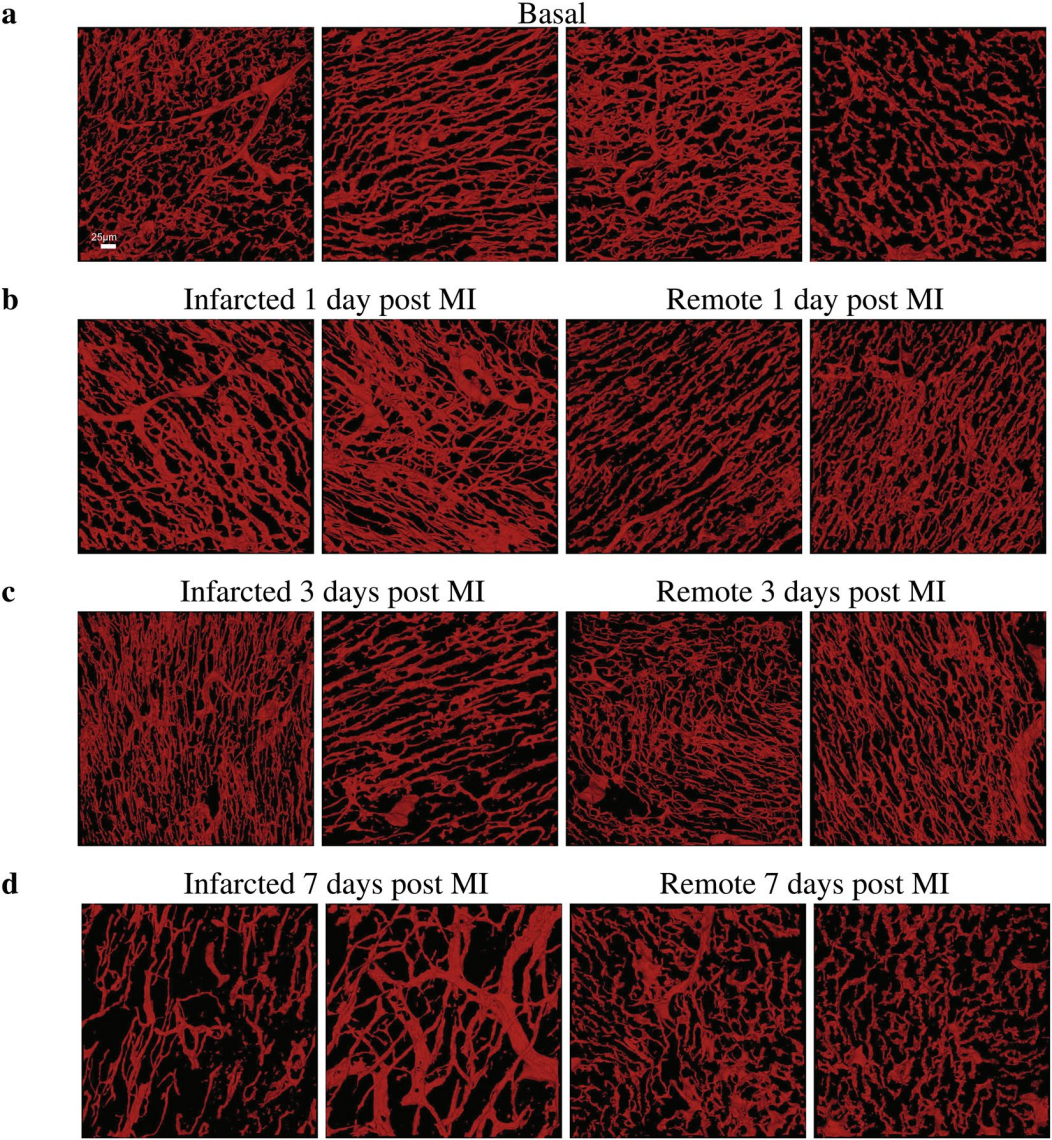
Representative 3D reconstructions of the cardiac microvasculature from tissues at different pathophysiological conditions. **(a)** Microvasculature reconstructed from tissues at basal conditions. **(b)** Microvasculature reconstructed from tissues at 1 day post MI. **(c)** Microvasculature reconstructed from tissues at 3 days post MI. **(d)** Microvasculature reconstructed from tissues at 7 days post MI. In panels (**b**–**d**) the first two volumes correspond to tissues from infarcted areas at the corresponding time-point after MI, while the third and fourth volumes to tissues from remote ones. Scale bar (25 *μm*) is the same for all images.

After the VE-Cadherin, as well as SMA^+^ cells, and nuclei are segmented, and the 3D microvasculature is reconstructed, the next step is to quantify the parameters that allow description of the characteristics of the microvasculature and assessment of infarction-related changes. Figure 2 provides representative 3D reconstructions of the microvasculature at different conditions under investigation (basal, infarcted and remote at 1, 3, and 7 days following MI respectively) used for the analysis. Table 1 provides a detailed list of the parameters extracted to describe the complex microvascular network described in this and following sections. Statistical analysis of the parameters is performed to identify vital changes that occur at different stages following MI.

**Table 1:** List of the parameters (mean ± standard deviation) that are extracted automatically for the characterization of the cardiac microvasculature, its interactions with SMA^+^ cells and remodelling due to MI. I1MI, I3MI, and I7MI, refer to infarcted areas at 1 day, 3 and 7 days post MI respectively. Similarly, R1MI, R3MI, and R7MI refer to the corresponding remote areas.

	Basal	I1MI	I3MI	I7MI	R1MI	R3MI	R7MI
**Fractal-Based Metrics**							
1. Fractal Dimension	2.22 ± 0.03	2.26 ± 0.05	2.22 ± 0.04	2.15 ± 0.04	2.24 ± 0.04	2.23 ± 0.03	2.22 ± 0.05
2. Lacunarity (× 10^−2^)	76.92 ± 5.15	72.75 ± 3.85	78.08 ± 5.52	83.98 ± 2.96	76.33 ± 4.58	73.64 ± 2.73	76.1 ± 4.03
3. Succolarity (× 10^−2^)	0.33 ± 0.14	0.49 ± 0.23	0.28 ± 0.13	0.19 ± 0.11	0.32 ± 0.16	0.3 ± 0.16	0.3 ± 0.2
**Minkowski-Based Metrics**							
4. Vascular Volume Density (%)	6.75 ± 0.85	8.9 ± 2.82	6.46 ± 1.06	5.04 ± 0.92	7.21 ± 1.34	6.14 ± 1.29	6.64 ± 1.83
5. Surface Area Density (× 10^−3^) (*μm*^2^/*μm*^3^)	48.36 ± 7.05	55.06 ± 12.86	46.45 ± 6.95	27.15 ± 4.05	48.2 ± 6.25	47.1 ± 6.62	45.04 ± 11.28
6. Breadth Density (× 10^−3^) (*μm*/*μm*^3^)	1.47 ± 0.29	1.43 ± 0.27	1.42 ± 0.28	0.64 ± 0.11	1.37 ± 0.19	1.52 ± 0.23	1.27 ± 0.32
7. Euler-Poincaré Characteristic Density (1/*μm*^3^)	−4.32 ± 1.78	−4.84 ± 3.1	−3.36 ± 1.38	−0.05 ± 1.49	−3.5 ± 1.62	−4.06 ± 1.69	−3.56 ± 2.64
8. Capillary Volume Density (%)	6.07 ± 0.9	8.04 ± 2.63	5.7 ± 1	4.34 ± 0.85	6.55 ± 1.17	5.43 ± 1.22	5.7 ± 1.97
9. Capillary Surface Area Density (× 10^−3^) (*μm*^2^/*μm*^3^)	46.94 ± 7.15	53.47 ± 12.69	44.5 ± 7.15	25.34 ± 4	46.62 ± 6.19	45.11 ± 6.63	42.5 ± 11.91
**Graph-Based Metrics**							
10. Vascular length density (× 10^−3^) (*μm*/*μm*^3^)	3.51 ± 0.85	4.13 ± 1.43	3.51 ± 0.82	1.65 ± 0.3	3.37 ± 0.58	3.54 ± 0.74	3.18 ± 1.21
11. Vascular surface density (× 10^−3^) (*μm*^2^/*μm*^3^)	38.87 ± 6.87	51.79 ± 18.71	38.05 ± 7.24	24.37 ± 4.63	40.35 ± 7.75	36.94 ± 8.29	36.94 ± 13.86
12. Vascular volume density (× 10^−2^) (*μm*^3^/*μm*^3^)	3.94 ± 0.66	6.16 ± 2.75	3.92 ± 0.93	3.33 ± 0.87	4.48 ± 1.3	3.59 ± 1.11	3.94 ± 1.68
13. Vascular segment radius (*μm*)	2.65 ± 0.31	2.91 ± 0.33	2.62 ± 0.3	3.25 ± 0.29	2.79 ± 0.33	2.48 ± 0.26	2.74 ± 0.26
14. Vascular segment length (*μm*)	18.85 ± 2.64	16.09 ± 2.83	17.65 ± 2.22	15.74 ± 2.77	18.15 ± 2.96	19.89 ± 3.21	17.59 ± 2.77
15. Vascular segment surface (*μm*^2^)	215.3 ± 49.14	200.75 ± 35.27	193.63 ± 27.74	231.67 ± 35.76	218.53 ± 42.92	207.73 ± 33.87	206.2 ± 34.89
16. Vascular segment volume (*μm*^3^)	224.15 ± 71.59	234.5 ± 54.3	200.87 ± 46.54	313.63 ± 59	241.77 ± 63.37	200.19 ± 42.08	220.22 ± 50.23
17. Tortuosity (*μm*/*μm*)	1.39 ± 0.01	1.38 ± 0.02	1.4 ± 0.03	1.38 ± 0.04	1.38 ± 0.02	1.39 ± 0.02	1.39 ± 0.03
18. Vascular segments (× 10^5^)^a^	1.91 ± 0.6	2.77 ± 1.48	2.02 ± 0.57	1.09 ± 0.3	1.91 ± 0.49	1.86 ± 0.68	1.9 ± 0.97
19. Vessels of diameter < =6.9 (*μm*) (%)	94.56 ± 2.94	90.14 ± 5.45	93.02 ± 4.1	83.92 ± 6.01	92.14 ± 5.02	95.56 ± 2.93	92.94 ± 3.03
20. Vessels of diameter between 6.9 and 8.2 (*μm*) (%)	3.01 ± 1.46	4.88 ± 2.02	3.54 ± 1.83	8.46 ± 2.31	3.92±2.06	2.21 ± 1.38	3.9 ± 1.52
21. Vessels of diameter >8.2 (*μm*) (%)	2.42 ± 1.57	4.98 ± 3.56	3.44 ± 2.62	7.62 ± 4.21	3.94 ± 3.07	2.23 ± 1.68	3.16 ± 1.74
22. Branching nodes (× 10^4^)^a^	10.14 ± 3.43	14.79 ± 8.12	10.74 ± 3.33	5.48 ± 1.58	9.94 ± 2.71	9.79 ± 3.84	9.85 ± 5.31
23. Blind-ends/sprouts (×10^4^)^a^	5.08 ± 1.42	7.47 ± 3.93	5.37 ± 1.3	3.44 ± 0.89	5.64 ± 1.47	4.95 ± 1.75	5.49 ± 2.38
24. Branching nodes^b^	30.02 ± 4.03	35.1 ± 6.26	31.29 ± 4.76	32.91 ± 5.61	30.54 ± 5.22	28.58 ± 4.57	31.11 ± 5.37
25. Blind-ends/sprouts^b^	18.22 ± 5.81	19.46 ± 4.52	16.52 ± 3.41	20.47 ± 4.18	18.99 ± 4.59	16.92 ± 4.32	21.04 ± 6.56
SMA^+^ **related metrics**							
26. Vessels covered with SMA (%)	80.05 ± 11.57	67.32 ± 19.45	69.34 ± 7.81	54.03 ± 9.82	73.82 ± 9.71	74.5 ± 15.59	78.01 ± 14.33
27. SMA^+^ layer thickness (*μm*)	2.43 ± 1.15	2.32 ± 0.77	4.13 ± 2.4	10.23 ± 1.76	2.7 ± 0.69	2.75 ± 0.8	3.31 ± 1.37
28. Damage Index	0.01 ± 0.01	0.01 ± 0.01	0.02 ± 0.02	0.14 ± 0.06	0.01 ± 0.01	0.02 ± 0.01	0.02 ± 0.02
29. Myofibroblasts (× 10^4^)^a^	1.04 ± 0.51	0.9 ± 0.56	0.96 ± 0.42	2.12 ± 0.77	0.64 ± 0.32	0.77 ± 0.34	0.67 ± 0.44
30. SMA^+^ perivascular cells^b^	31.71 ± 11.34	24.58 ± 12.6	24.83 ± 8.48	45.72 ± 17.49	30.95 ± 6.76	22.68 ± 7.48	26.19 ± 10.53
**Efficiency in oxygen diffusion**							
31. Maximal Extravascular Distance (*μm*)	27.35 ± 6.76	28.29 ± 4.52	33.72 ± 6.8	46.22 ± 8.26	30.37 ± 5.13	28.32 ± 3.71	29.69 ± 4.42
32. Median Extravascular Distance (*μm*)	14.57 ± 3.56	15.07 ± 2.38	17.92 ± 3.58	24.51 ± 4.35	16.16 ± 2.7	15.08 ± 1.95	15.8 ± 2.33
33. Capillary Density^c^	1236.16 ± 714.07	1226.84 ± 567.85	1101.16 ± 525.2	394.24 ± 211.98	1091.95 ± 452.94	1053.55 ± 781.83	1059.93 ± 514.7
34. Intercapillary Distance (*μm*)	16.91 ± 2.08	16.93 ± 1.84	17.44 ± 2.46	25.54 ± 4.44	17.68 ± 1.62	17.17 ± 1.85	19.51 ± 3.64
35. Diffusion Distance (*μm*)	9.77 ± 1.43	9.1 ± 1.38	9.72 ± 1.17	14.07 ± 1.38	9.83 ± 0.83	9.63 ± 1	10.55 ± 2.17
**Additional cell-related metrics**							
36. Endothelial cells (× 10^5^)^d^	22.85 ± 6.83	19.19 ± 5.19	19.36 ± 3.82	18.31 ± 5.97	19.18 ± 4.5	19.17 ± 3.74	15.12 ± 2.76
37. Endothelial cells^b^	44.14 ± 8.53	40.93 ± 8.16	35.97 ± 5.78	55.47 ± 16.91	40.44 ± 6.09	33.1 ± 4.75	33.13 ± 7.98

^a^Number per *mm*^3^ of tissue.

^b^Number per *mm* vascular length.

^c^Number per *mm*^2^ of tissue.

^d^Number per *mm*^3^ vascular volume.

Parameters based on fractal analysis and on the Minkowski Functionals (MF) are used for describing the microvasculature as a complete network, and thus, identifying its alterations after MI at a structure-global level. Fractal analysis accounts for the multiscale properties of structures that can be self-similar, such as the vascular networks^33^, and it is powerful tool in cardiology^34^. Here, as in^30^, we used three fractal parameters calculated directly from the 3D volumes; fractal dimension, lacunarity and succolarity. Fractal dimension provides an estimation of the morphological complexity of structures. Lacunarity describes the heterogeneity in the distribution of gaps sizes. Gaps in the case of cardiac tissue represent non-vascularized areas. Succolarity indicates the capacity of a fluid, i.e. blood in the case of the microvasculature, to flow through the structure. On the other hand, the Minkowski functionals (MF)^35^ are morphological descriptors (volume, surface area, mean integral of breadth and Euler-Poincaré characteristic) that encompass standard geometric and topological (connectivity) properties of structures. Thus, a combination of fractal and Minkowski-based parameters, allows obtaining complementary information regarding the microvascular networks.

**Figure 3:**
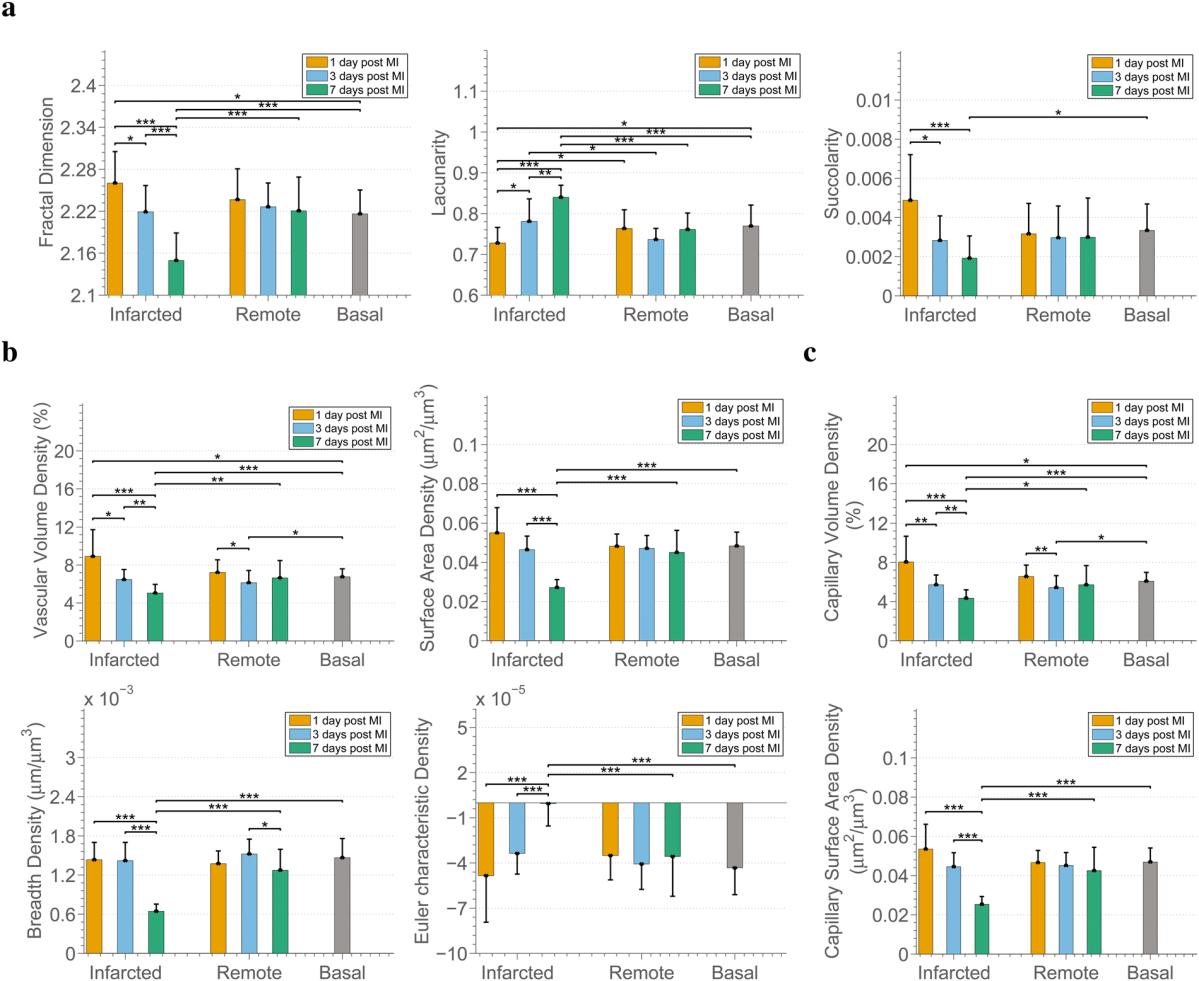
Myocardial infarction decreases 3D complexity, integrity, and connectivity of the coronary microvasculature while it alters heterogeneity in the distribution of non-vascularized areas and capacity for blood flow. **(a)** 3D fractal-based parameters; Fractal Dimension, Lacunarity, and Succolarity. **(b)** Parameters based on the Minkowski functionals calculated for complete microvasculature (capillaries, arterioles, venules); Vascular Density, Surface Area Density, Breadth Density, Euler-Poincaré Characteristic Density. **(c)** Parameters based on the Minkowski functionals calculated for capillaries exclusively; Capillary Volume Density, Capillary Surface Area Density. In all figures of the manuscript, the bars show mean values, while error bars represent standard deviation. Furthermore, *, ** and *** represent p-value < 0.05, 0.01 and 0.001 respectively. The p-values were calculated by means of Wilcoxon rank-sum tests and corrected with the Benjamini-Hochberg procedure for multiple testing. The number of samples compared is 18 per tissue condition.

The fractal parameters demonstrate significant differences in terms of multi-scale characteristics of the microvascular networks in the infarcted areas, over time (Fig. 3a). In particular, when comparing infarcted areas at 1, 3, and 7 days, we noted that the morphological complexity (fractal dimension) and capacity of blood flow (succolarity) of the microvasculature decreased, while the heterogeneity in the distribution of the non-vascularized areas (lacunarity), increased. The reverse relationship between morphological complexity and heterogeneity has been observed in earlier studies^30,36^. Moreover, the distribution of non-vascularized areas affects blood flow. Thus, higher heterogeneity, as observed here, is expected to result in higher heterogeneity in blood flow within the tissue, and could therefore denote the existence of regions with lower perfusion. Furthermore, complexity and heterogeneity in the infarcted areas at 1 and 7 days are statistically different than in the corresponding remote areas (1 and 7 days respectively). In terms of all multi-scale characteristics, the differences of the microvasculature from infarcted areas at 7 days post MI are significant compared with that at basal conditions. It is worth also noting that at infarcted areas 1 day post MI the tendencies compared with basal conditions are opposite to 7 days: higher morphological complexity and blood flow instead of lower, and lower lacunarity instead of higher. This could be linked to the first response of the system to MI and the poorer contractility of cardiomyocytes at day 1. Moreover, in contrast with the changes observed in microvascular networks from infarcted areas, no significant differences are found in the remote areas over time and compared with the basal case.

The reduction in the morphological complexity of the microvasculature in infarcted areas can be further explained by the progressive loss of microvascular volume that occurs in the infarcted areas as revealed by the decreased vascular volume density (Fig. 3b) observed in those areas. Furthermore, the microvasculature appears reduced in the infarcted areas, with less breadth and smaller surface area for the exchange of oxygen and nutrients, while it is progressively converted from a strongly connected network to a less connected one (progressively increasing Euler-Poincaré characteristic). The reduced connectivity and volume are related to the lower capacity of blood flow and probably to its heterogeneity described earlier. An unexpected observation is the significant decrease of the microvascular volume density at remote areas at 3 days post MI compared with remote at 1 day post MI and basal. Further exploration of the lost vascular volume (capillary volume or arteriole/venule volume) led to the observation that the loss is associated with the loss of capillary volume (Fig. 3c). Lastly, as with the fractal parameters, in terms of all metrics the infarcted area 7 days post MI presents significant differences with basal areas, as well as with corresponding remote areas.

### Myocardial infarction promotes microvascular angioadaptation

**Figure 4:**
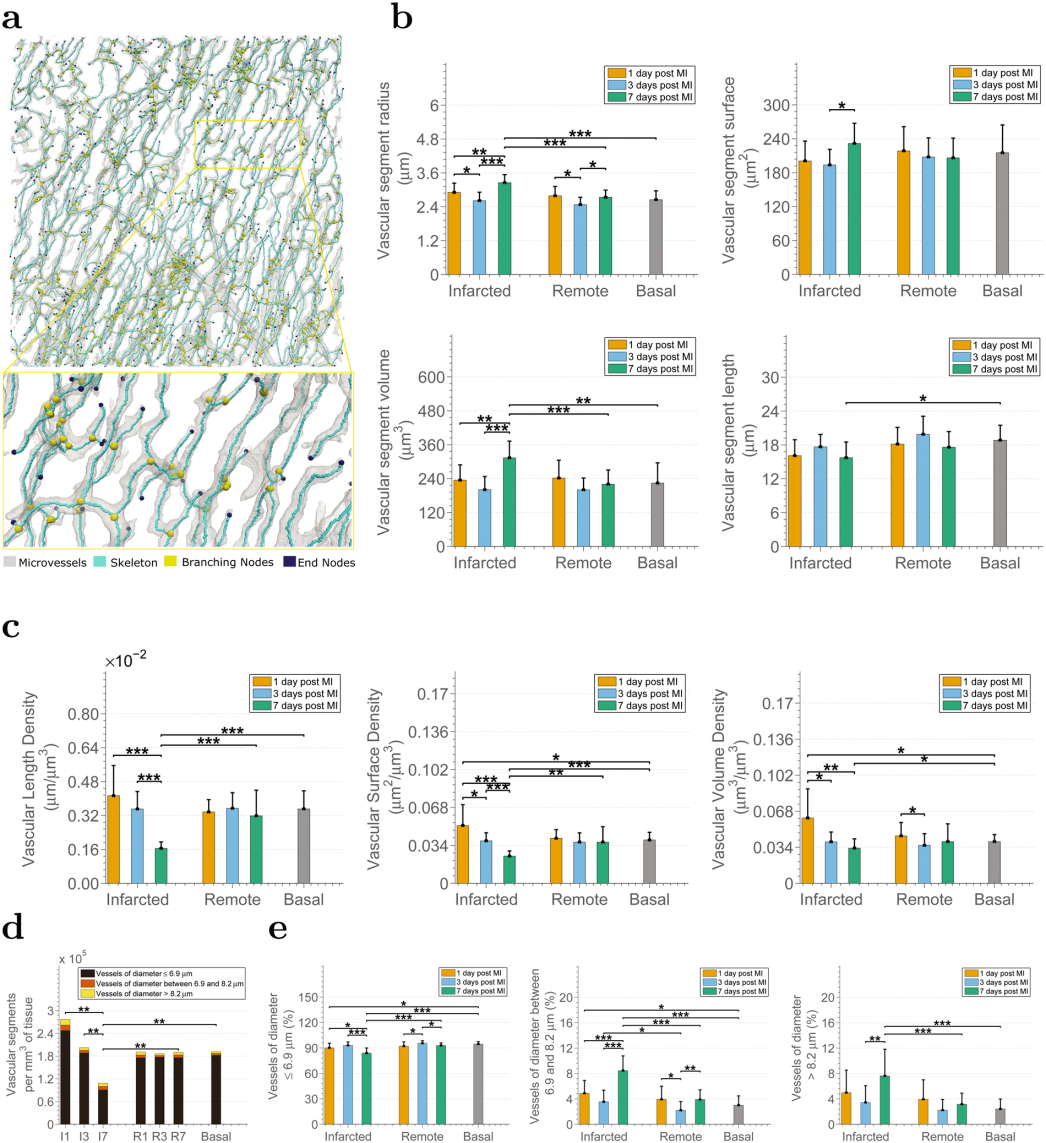
Microvascular angioadaptation occurs in response to MI. (**a**) Example graph-based representation of a microvascular structure. Each microvessel, i.e. segment from branching node to branching/ending node, is considered as a tube of constant radius. **(b)** Analysis of the characteristics of the microvessels in the pig infarcted heart in terms of vascular segment radius, vascular segment length, vascular segment surface, and vascular segment volume. **(c)** Analysis of vascular length density, vascular surface density, and vascular volume density. In panels **(d)** and **(e)**, changes in the number and percentages of microvessels in the infarcted pig heart according to their radius and to the physiological condition of the tissue. In particular, **(d)** analysis of the number of microvascular segments per *mm*^3^ of tissue. **(e)** Analysis of the percentage of vascular segments with radius smaller than 6.9 *μm*, with radius between 6.9 and 8.2 *μm* and with radius larger than 8.2 *μm*. I1, I3, I7, R1, R3, R7 stand for infarcted areas and remote areas at 1, 3, 7 days post MI respectively. Note that statistical hypothesis testing in **(d)** refers to the comparison of the overall number of vascular segments i.e. the sum of the three categories.

Metrics at the structure-global level provide us with paramount information regarding the whole microvascular plexus, but they do not provide insights into the angioarchitecture, i.e. the morphology and arrangement of the individual microvessels that form the microvasculature, on which the functional properties of microcirculation and their modelling critically depend^37^. For this reason, we perform a quantitative analysis at the segment level using the 3D graph-based representation of the microvasculature (Fig. 4a), complemented with information regarding the radius, length, surface and volume of every segment (see Methods).

The most profound changes are observed at infarcted areas 7 days after MI. By examining Fig. 4b it is obvious that the microvascular segments of those regions present larger radius than those of tissues from infarcted areas at earlier time-points, remote areas at 7 days post MI and at basal conditions, despite the reduction of the overall microvasculature described in the previous subsection. This results in segments with significantly larger volume than segments from the other areas, while their surface area is significantly larger than that of segments from infarcted areas at 3 days post MI and their length is shorter than segments from basal conditions. In contrast, the microvessels in both the infarcted and remote areas 3 days post MI become significantly smaller in terms of radius compared to those of the infarcted areas from earlier and later time-points under investigation, indicating a possible vasoconstriction on this time-point. The radius of microvessels from infarcted and remote tissue 1 day following MI, as well as from remote areas 7 days post MI, seem to remain unaffected. No significant difference among any tissue categories is observed in terms of tortuosity and the relative plot was, therefore, omitted. However, the corresponding mean and standard deviations are provided in Table 1.

Length, surface and volume density (Fig. 4c) calculated by means of the graph-based representation of the microvasculature confirm the microvascular changes revealed by the corresponding Minkowski-based metrics (breadth, surface and volume density). This fact demonstrates that the latter are sufficient in describing those characteristics of microvascular networks and it is therefore feasible to avoid exhaustive extraction of centerlines when characteristics of the individual microvessels are out of interest.

Subsequently, segments were divided into three classes based on the morphological data of^13^ in order to study the size of microvessels at each tissue condition. The maximum diameters found in the left ventricle of the pig heart according to the published data are 7.3 *μm* for capillaries fed directly by arterioles (*C*_*oa*_), 8.2 *μm* for capillaries drained directly by venules (*C*_*ov*_), and 6.9 *μm* for cross-connecting capillaries (*C*_*cc*_) and capillaries connected to *C*_*oa*_ and *C*_*ov*_ types (*C*_*oo*_). Thus, the three classes created are (i) segments with diameter smaller or equal to 6.9 *μm* corresponding to *C*_*cc*_ and *C*_*oo*_ capillaries, (ii) segments with diameter between 6.9 and 8.2 *μm* corresponding to *C*_*oa*_ and *C*_*ov*_ capillaries, and (iii) segments with diameter larger than 8.2 *μm* representing arterioles or venules.

A gradual decrease in the overall number of microvascular segments per *mm*^3^ of tissue is observed at the infarcted areas (Fig. 4d). The decrease becomes profound and statistically significant at 7 days post MI compared with all other conditions. The reduced number of segments explains the reduced morphological complexity, vascular volume density, surface area and breadth density of this area expressed by the fractal dimension and the Minkowski metrics (Fig. 3). Furthermore, a higher number of vascular segments can be also observed in the infarcted area 1 day post MI compared to the basal tissue. This difference, although not significant, matches the higher density and morphological complexity on this tissue category described in the previous subsection (Fig. 3).

Looking further into the percentage of vessels in each radius range (Fig. 4e) the percentage of microvessels with diameter larger than 8.2 *μm*, i.e. arterioles/venules, and “medium”-sized capillaries of the categories *C*_*oa*_ and *C*_*ov*_ (6.9 and 8.2 *μm*) are increased in infarcted areas 7 days post MI. On the contrary, the smallest vessels (*C*_*cc*_ and *C*_*oo*_) are reduced compared to the healthy tissue, infarcted tissue at earlier stages after MI as well as with the corresponding remote area. This fact demonstrates a regression of smaller microvessels and probably dilation or even arterialization of the remaining vessels in infarcted tissues from 7 days following MI. The smallest microvessels from infarcted tissue 1 day post MI are significantly decreased as well, while the percentage of capillaries *C*_*oa*_ and *C*_*ov*_ (6.9 and 8.2 *μm*), is increased when comparing to microvessels belonging to basal tissue, which implies a possible vasodilation effect. Interestingly, at remote areas 3 days post MI the number of smallest capillaries is increased compared with the remote areas at other time-points, and in contrast, the percentage of “medium”-sized vessels is reduced.

**Figure 5:**
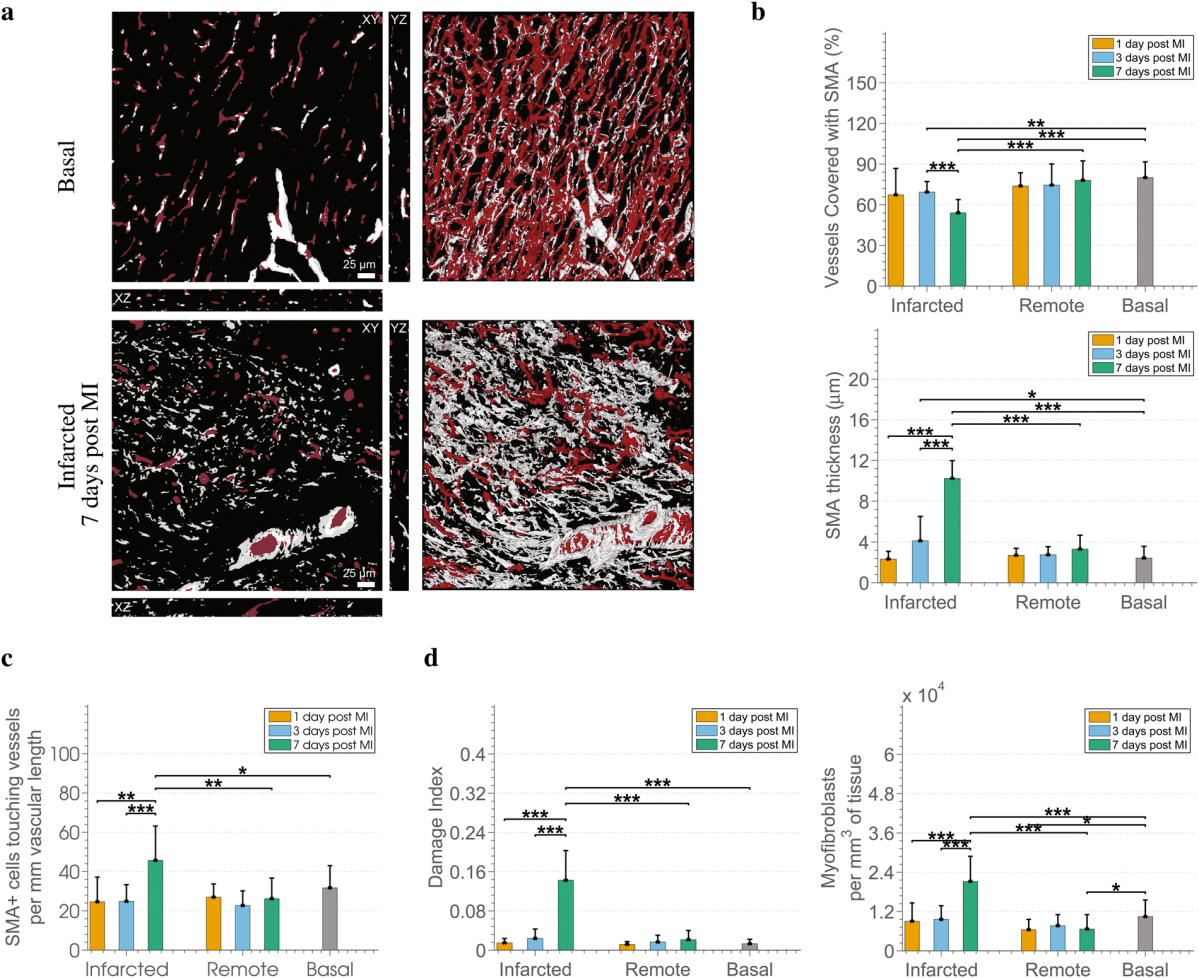
Changes in SMA^+^ perivascular cells and myofibroblasts in the aftermath of MI. **(a)** Example of 3D reconstructions of the microvasculature (red) merged with the segmentation of the SMA^+^ cells channel (grey) in a basal and an infarcted 7 days post MI case. **(b)** Changes in SMA^+^ related metrics, i.e. the percentage of vessels with a SMA^+^ coat along with the corresponding thickness of the coat. **(c)** Analysis of the number of SMA^+^ cells of which the SMA^+^ coat covering the microvessels consists. **(d)** Analysis of the cardiac tissue by means of indicators of tissue fibrosis, i.e. the Damage Index (percentage of SMA^+^ volume that are not associated with vessels), and number of myofibroblasts per *mm*^3^ of tissue.

Information from the α-SMA channel and its relation to the microvasculature (Fig. 5a) is quantified through an additional module of the analysis pipeline in order to study perivascular cells stained by α-SMA, i.e. pericytes and smooth muscle cells. We are particularly interested in the coating of microvessels by layer(s) of α-SMA expressing cells as this could be an indicator of vessel maturation^38,39^ and denote the end of a plasticity window for intervention^40^. A significantly lower percentage of vessels coated with α-SMA^+^ cells is observed in infarcted areas 3 and 7 post MI compared to the basal tissue as well as to the corresponding remote area for the second case (Fig. 5b). However, the layers of α-SMA^+^ perivascular cells on those days, i.e. 3 and 7 days post MI, are significantly thicker than in the basal case as shown in the same panel. Furthermore, the thick layer of α-SMA^+^ at the infarcted area 7 days post MI consists of significantly more cells than in the infarcted areas of previous time-points, the basal and remote area 7 days post MI as indicated by the increased number of α-SMA^+^ perivascular cells per *mm* of vascular length (Fig. 5c). Thus, we have a proliferation of the perivascular α-SMA^+^, and not a dilation of the cells.

We also noticed, mainly in infarcted areas, α-SMA^+^ regions that were not associated with microvessels, i.e. they were not localized in the perivascular layers of microvessels. Given that myofibroblasts are non-vascular cells that produce extracellular matrix indispensable for healing after MI and have contractile properties related to their expression of α-SMA^41^, we considered those α-SMA^+^ regions corresponding to myofibroblasts. We further complemented their characterization by their concomitant expression of other markers, such as platelet-derived growth factor receptor beta (PDGFRB)^42^, vimentin and collagen I^43^ (Supplementary Fig. S4). We then decided to extract quantitative information regarding the myofibroblasts based on the α-SMA^+^ staining. For this purpose, a damage index was defined for the tissue as an estimator of the presence of myofibroblasts. Precisely, the index is given by the ratio of the volume of myofibroblasts over the volume of all α-SMA^+^ regions. Furthermore, the number of the myofibroblasts is quantified as the number of simultaneously stained nuclei by Hoechst and α-SMA.

Damage index (Fig. 5d) demonstrates a non-significant increase in the presence of myofibroblasts in infarcted tissues at the first time-points after MI (1 and 3 days) followed by a significant increase at the later time point (7 days post MI). This observation is in accordance with findings in the healing human tissue^44^, and rat tissue after MI^45^. A lack of increase in the damage index in the remote areas compared to the basal case denotes no increase of myofibroblasts, and thus, no inappropriate deposition of extracellular matrix in remote areas^41^. As far as the absolute number of myofibroblasts per *mm*^3^ of tissue is concerned, the trends presented in infarcted areas are similar with that of the damage index. However, additionally a decrease of myofibroblasts in remote areas at 1 and 7 days post MI compared with the basal case can be observed.

### 3D microvascular characteristics predict the healthy or diseased status of the cardiac tissue

The quantitative information about the cardiac microvasculature and SMA^+^ cells obtained by our pipeline was evaluated with the task of predicting the condition of previously unseen tissue based on the characteristics of the microvascular bed. Towards this aim, we incorporated the parameters at structure level, at segment level as well SMA^+^ related metrics (metrics 1–28 of Table 1) into a classification scheme using three different classifiers; (i) K-nearest neighbours classifier (Knn), (ii) Support Vector Machines (SVM), and (iii) Adaboost^46^. In all cases, 9-fold cross validation repeated 10 times was used. Accuracy rates (%) are presented in Table 2.

**Table 2:** Accuracy (%) in classifying the distinct vascular patterns using different classifiers: (1) Knn, (2) SVM, (3) Adaboost, and 9-fold cross-validation, repeated 10 times. Metrics of 1 − 28 of Table 1 are used as features for performing the classification.

Classifier	1	2	3	Classifier	1	2	3
**Infarcted - Basal**	77	81	74	**Remote - Basal**	68	72	75
I1MI - Basal	82	75	59	R1MI - Basal	61	70	61
I3MI - Basal	66	77	47	R3MI - Basal	65	63	57
I7MI - Basal	100	100	94	R7MI - Basal	63	59	70
**Infarcted over time**	75	89	72	**Remote over time**	48	50	40
I1MI - I3MI	71	84	64	R1MI - R3MI	61	66	57
I1MI - I7MI	100	100	97	R1MI - R7MI	53	60	56
I7MI - I3MI	93	97	93	R7MI - R3MI	56	64	56
**Infarcted - Remote**	67	71	56				
I1MI - R1MI	53	68	69				
I3MI - R3MI	62	60	42				
I7MI - R7MI	97	100	88				

The proposed approach achieves high accuracy in the range 74–81% in differentiating between infarcted and basal tissue, as well as in recognizing the day after MI that the infarcted tissue belongs to (72–89%). The accuracy in classification drops to levels of 68–75% and 56–71% in differentiating between remote and basal tissue, and between remote and infarcted tissue respectively. This is expected taking into account the lack of significant differences in the majority of metrics between those categories. It is worth noting that, for the same reason, the worst levels of accuracy in classification (40–50%) are achieved in terms of recognizing which day following MI the remote tissue belongs to.

### Structural changes of the microvasculature support altered oxygen diffusion in the post-infarcted cardiac tissue

The diffusion of oxygen from the microvascular network to the tissue critically depends on the density and the arrangement of the microvascular bed^47^. Simple metrics, such as capillary density, intercapillary distance, diffusion distance as derived from vascular length density, and 3D extravascular distances (EDs), have been proposed in the literature as indices of oxygen diffusion, with the latter having been proven more efficient^48^.

**Figure 6:**
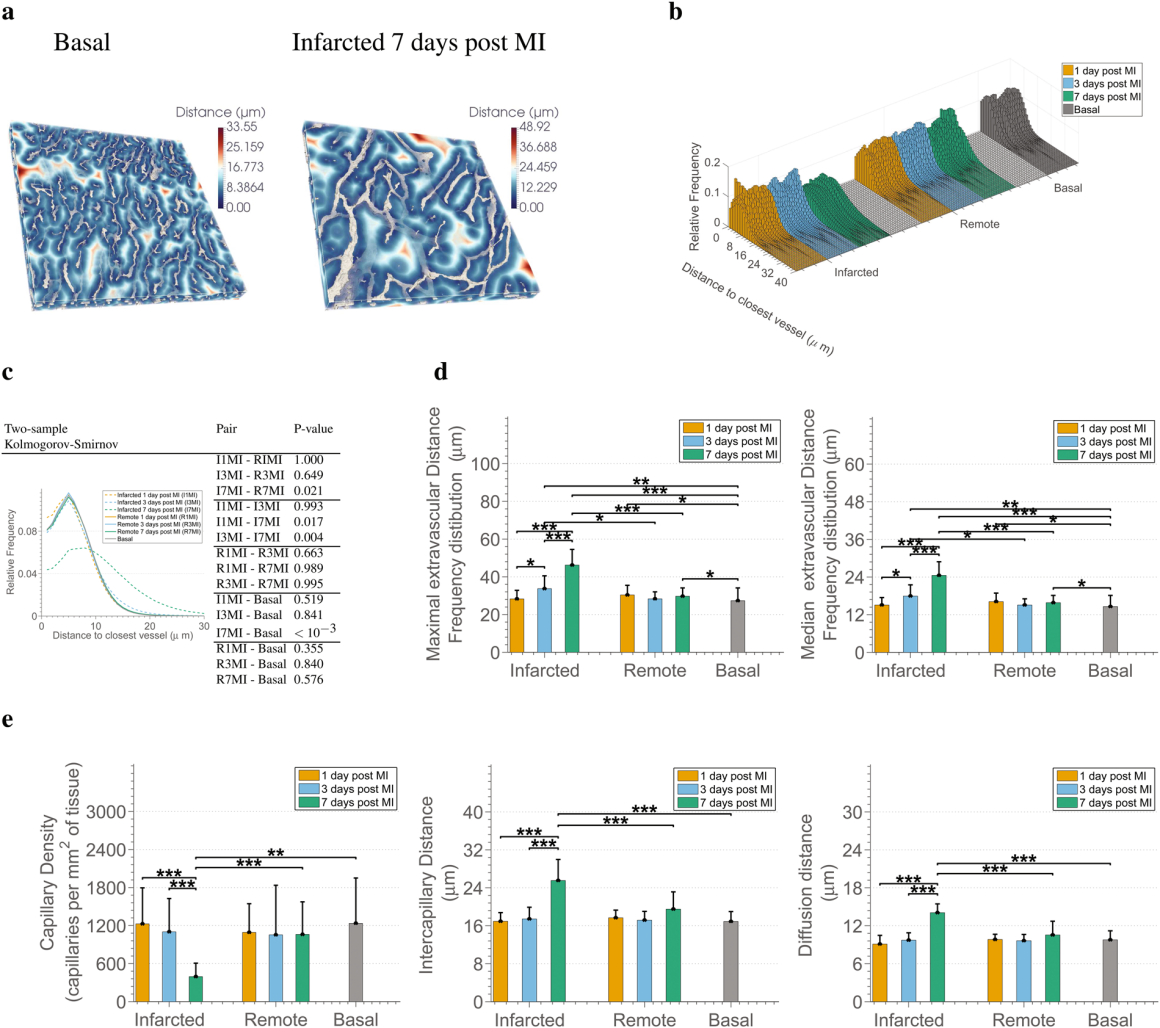
Capillary diffusion distances support altered oxygen diffusion in the infarcted cardiac tissue. **(a)** Example of 3D extravascular distance maps for a basal and a 7 days post MI case. **(b)** Histograms of the extravascular diffusion distances for all volumes of the dataset colour-coded according to the tissue condition. **(c)** Kolmogorov-Smirnov statistical tests for identifying significant differences in the mean relative frequencies of diffusion distances between the different tissue categories. **(d)** Analysis of the maximum and median extravascular diffusion distances at the different tissue conditions based on all volumes of the dataset. **(e)** Analysis of metrics traditionally related to oxygen-diffusion distances, i.e. capillary density, and inter-capillary distance calculated from 2D slices of the 3D volume, and of the diffusion distance calculated by using the length density, instead of the detailed 3D maps of panel.

In this work, 3D maps of EDs were calculated by getting the distance of every point to the closest vessel (Fig. 6a) and they were used to calculate the relative frequency distributions (Fig. 6b). From the comparative plot, the most striking difference that we can observe among the different tissue conditions is a long-tail in the distribution of EDs at the infarcted areas 7 days following MI. This tail implies longer diffusion distances, which are associated with longer diffusion times, and thus worst oxygenation^47^. Two sample Kolmogorov-Smirnov tests were used to compare the mean relative frequencies of each tissue category (Fig. 6c). The outcome verified the statistical significance of the difference in the shape of the distributions. The median and maximal EDs, calculated as the 50% and 95% quartile respectively^48^, also confirm significantly increased diffusion distances in the infarcted areas 7 days post MI. However, significant differences in both metrics in the infarcted areas 3 days following MI, compared to the basal and corresponding remote tissue, highlight that the increase in the EDs had already started in the infarcted areas from day 3 following MI. Moreover, the EDs at remote areas at 1 and 7 days after MI are also affected and are significantly different from that of the basal tissue.

EDs calculated directly from the complete 3D volumes are expected to be more accurate than capillary density and intercapillary distance which are calculated in 2D, or the diffusion distance which is derived from the vascular length density. However, for the sake of completeness and for facilitating comparison with past and future works, these metrics are provided in Fig. 6d. According to the plots, the capillary density is affected and reduced in infarction, with the reduction becoming significant at 7 days post MI in infarcted area. On the other hand, the intercapillary distance which is associated with an inverse relationship with capillary density, is increased in the infarcted areas at 7 days following MI. The diffusion distance calculated directly from the length density follows the same trend as the EDs calculated from the 3D distance maps, although an underestimation of the distances can be observed.

**Figure 7:**
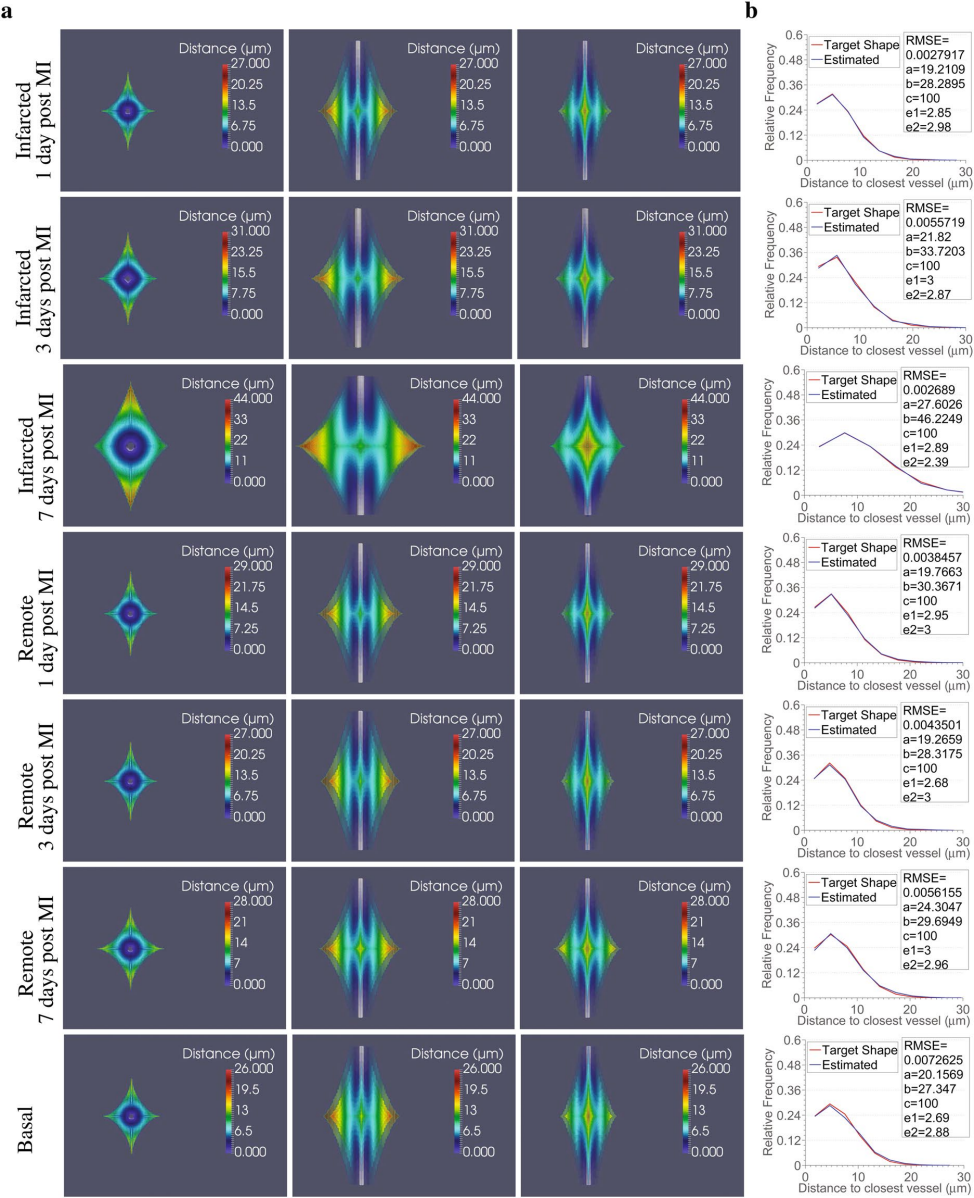
3D super-ellipsoids are adequate geometrical models to represent the capillary diffusion area. **(a)** 3D super-ellipsoids estimated to best fit the mean distributions of extravascular distances extracted from histograms of 10 bins for the different tissue conditions (one per row; basal, infarcted and remote 1, 3, 7 days following MI) from x-y (first column), x-z (second column) and y-z views (third column). The tissue points inside the 3D shapes are pseudo-coloured according to the distance to the central capillary (represented with grey in all sub-figures). The radius of the central capillary for each tissue condition was set equal to the minimum extravascular distance found for the particular case. **(b)** Accuracy of the fitting between the mean distributions of extravascular distances for the different tissue conditions (target represented with red) and the distributions produced using the estimated 3D super-ellipsoids of panel a to best fit them (represented with blue). RMSE stands for the root mean square error, while *a*, *b*, *c*, *e*_1_, *e*_2_ are the estimated parameters that define the 3D super-ellipsoids. In particular, *a*, *b*, *c*, are the scale factors for axis *x*, *y* and *z* respectively, and *e*_1_, *e*_2_ control the shape of the super-ellipsoid.

Lastly, we contrasted the mean relative frequency distributions of 3D EDs for each tissue category with the ones that would be provided by equivalent Krogh cylinder models^49^. The Krogh cylinder model assumes the supply of oxygen by every capillary to a cylindrical region whose radius equals half the intercapillary distance. As a result, the relative frequency distributions in the Krogh cylinder model are monotonically increasing to the maximum distance to the closest vessel (Supplementary Fig. S3) and are far from describing the distributions observed in the cardiac tissue studied in this work. In an effort to identify a geometric pattern that would describe the area of capillary diffusion so that it closely matches the relative frequency distributions observed in our volumes, we used a super-ellipsoid model. Super-ellipsoids allow description of a variety of shapes, ranging from cylinders (such as in the Krogh model) to more complex ones, by varying only a small number of parameters (see Methods). Thus, we formulated a question of finding a set of parameters that would define super-ellipsoids that match simplified versions of our distributions (10 bins) as an error minimization problem between the observed relative frequency distributions and the ones corresponding to different super-ellipsoids (Fig. 7). Root mean squared error in the range of 0.0027–0.0073 between the relative frequency distributions of extravascular distances in the super-ellipsoid models and the target ones demonstrate the superiority of the super-ellipsoid model over the Krogh cylinder model to represent the region of capillary diffusion in the cardiac tissue studied here.

### Changes in the microvasculature and in the diffusion of oxygen persist and intensify at later time-points post MI

We next analyzed, by means of our image analysis pipeline, the microvascular characteristics of porcine tissues from infarcted and remote areas 45 days post MI (Supplementary Fig. S5) to examine whether the observed microvascular alterations during the first week post MI persisted at later time-points and whether additional changes occurred. The results, which have been summarized in Supplementary Table S1 point to the persistent nature of the extensive microvascular remodelling that takes place 7 days post MI in infarcted areas together with a deterioration in certain metrics at 45 days post MI. Changes, which had been slightly observed at remote areas 7 days post MI, also intensified and became evident at remote areas 45 days post MI.

Both infarcted and remote areas at 45 days post MI present the same increasing or decreasing trends in those parameters which describe the microvasculature (fractal-, minkowski- and graph-based metrics) as the corresponding tissue 7 days post MI compared to earlier time-points post MI and basal conditions. There are several metrics, however, where increases or decreases are so prominent that statistically significant differences are observed between the tissues examined 7 and 45 days post MI. More precisely, infarcted areas at 45 days post MI have less vascular surface, breadth and length density, while they present a higher heterogeneity in distribution of non-vascularized areas compared to 7 days post MI. Concomitantly, there were fewer smaller and more medium and larger microvessels with increased intercapillary and diffusion distances at 45 days. Altogether this remodelling indicates worse vascular perfusion and oxygen supply in infarcted areas at late time-points. In the remote areas at 45 days similar changes were observed for the microvasculature which was characterized by a further loss of its morphological complexity, breadth and connectivity. These changes are accompanied again by enlarged vessels, reduction in smaller and increase in larger microvessels along with higher heterogeneity in the distribution of non-vascular areas as well as a trend to poorer diffusion. Moreover, the SMA^+^ perivascular layer is thinner, while the number of myofibroblasts decreases in both remote and infarcted areas compared to day 7 post MI. This suggests deceleration or termination of extracellular cardiac remodelling at this late stage.

## Discussion

The link between the anatomy of microcirculation and CVDs’ onset and progression had led to considerable efforts in unravelling the fine perturbations of the microvascular networks. This is particularly true in the case of MI were an amount of important works had been performed prior to our work^38,40,50–56^. However, there is a high variability among the quantification methods used in those studies, with each study reporting a different set of parameters to characterize the anatomy of microcirculation and some of them being based on the 2D analysis. Works more closely related to ours^56^ do not provide the wealth of data which are indispensable in describing the complications of MI, but are rather based on only a few traditional metrics.

This work was designed to deal with these shortcomings by allowing the in-depth study of all major characteristics of the microvasculature in 3D through a single pipeline. Our pipeline consists of modules that permit translation of image information to quantitative biological knowledge regarding (i) the multiscale and geometric properties of the microvasculature, (ii) topological characteristics and biologically important morphological information about the segments the microvascular structure consists of, (iii) the microstructural relationship with SMA^+^ cells, which are key players in tissue healing after MI (myofibroblasts), as well as in vessel maturation and stabilization (SMA^+^ perivascular cells), and (iv) diffusion efficiency of the microvascular bed and adequate geometric models of the capillary supply region. We show that each class of parameters offers complementary information in understanding the effects of MI and that it is indispensable to study all classes in order to understand changes to a system as complex as the microvasculature.

Furthermore, we provide a novel method for accurately reconstructing the complete microvasculature from VE-Cadherin-labelled endothelial junctions. This approach will enable the use of VE-Cadherin, an endothelial marker with high specificity even in infarction, to reconstruct microvessels and it will allow the simultaneous study of endothelial junctions, SMA^+^ and microvessels through the use of two fluorescent markers (VE-Cadherin, SMA^+^ cells) instead of three. The latter is noteworthy given that the number of fluorochromes that can be imaged simultaneously is limited^57^. Lastly, a simple, yet efficient, approach in distinguishing between capillaries and arterioles/venules was incorporated in the pipeline. The importance of identifying the type of microvessels is not solely limited to the definition of the 3D guidance map for filling (and pruning), but it is also crucial in the implementation of theoretical modelling approaches^58^. The approach could be further extended to separate arterioles from venules, given their morphological characteristics such as branching patterns and tortuosity.

Among the main findings is an intensive structural remodelling of the microvasculature at 7 days post MI, which persists and even deteriorates 45 days post MI, in combination with an abundant presence of myofibroblasts, a higher percentage of microvessels which lacked a SMA^+^ coating, but with the coated ones having a thick SMA^+^ layer. The latter might mark the end of the plasticity window for intervention^40^. By quantifying the effect of the structural remodelling in functional terms, we were able to identify a low capacity for blood flow (succolarity) and long diffusion distances which is linked with worse oxygenation. This might be related to the regression of vessels non-coated with SMA^+^ resulting in a less complex and connected network that, as a result, requires longer diffusion times. Moreover, despite the decrease in the capacity of blood flow at the tissue level, observed also at organ level (Supplementary Table S2) at infarcted areas 7 days post MI, the larger diameter of the individual microvessels and the SMA^+^ hyperplasia, together with increased number of endothelial cells per length (Table 1), likely reflects adaptation to persistent/chronic high flow in these remaining vessels. Interestingly, in terms of structural characteristics (density, size of microvessels) the infarcted area at 3 days post MI, seems closer to the basal one than at 1 or 7 days post MI. This could indicate a normalization of the effect of infarction 3 days following MI after a first inflammatory response at day 1 and, therefore, denote a time window for therapeutic intervention around day 3 post MI when the microvasculature is not structurally so different from the basal but functionally already starts the misadaptation as implied by the larger extravascular distances and higher heterogeneity in non-vascularized areas observed in infarcted areas 3 days post MI. Moreover, our results imply a connection between the appearance of constriction or dilation and the time which elapsed following MI. In particular, we observed a tendency for an initial dilation of the vessels in infarcted area at 1 day post MI, a subsequent constriction at 3 days, followed by possible pruning of microvessels, and maybe dilation/arterialization of the remaining microvessels at day 7. It is also worth noting that apart from the vasodilation effect the structural changes at infarcted areas at 1 day post MI are opposite to that of 7 days. Specifically, an increased number of microvessels, vascular density and complexity at infarcted areas at 1 day post MI were described. Thus, we cannot rule out angiogenesis (either sprouting or intussusceptive) at that time-point but it is clear that there is an overall loss of microvasculature afterwards, mostly of capillaries. In addition, as expected^59^, changes at remote areas are not significant or they are milder than the ones found in the infarcted areas, but they are not entirely absent as pathology progresses.

Despite the importance of our findings, there are some limitations to the current work. One of the strengths of this study is the choice of the highly translational porcine animal model. However, due to the cost and difficulty in handling large animals, we were able to use a limited number of subjects. Furthermore, the chosen ischaemia model might influence the microvascular changes described in this study. This means that the same changes may not be necessarily anticipated in other ischaemia models, such as permanent coronary artery ligation. This limitation could motivate future research as the presented approach paves the way to standardized analysis and future comparison of different experimental ischaemia setups. Another limitation of the present work is the loss of precise topological information at the organ level during tissue processing and confocal microscopy imaging. Also, in spite of the fully automated and un-biased analysis pipeline, human bias might be potentially introduced in the selection of the areas to be imaged by confocal microscopy and in the adjustment of the microscope settings. In addition, since no automated method can ensure 100% accuracy in terms of segmentation, the exact metric numbers might deviate slightly from reality, although the trends would remain uninfluenced given that the same method was applied to all images. Lastly, running the complete pipeline on large images can be computationally and time demanding, but this can be balanced by the fact that requires no human intervention, therefore, minimizing costs and labor requirements, while facilitating the calculation of complex metrics.

In conclusion, our fully automated image analysis pipeline permitted the accurate reconstruction of the 3D microvasculature and subsequent acquisition of novel, quantitative insights into its structure and changes at different stages after MI. The study was performed in the pig model, whose coronary microvasculature bears strong similarities to that of humans. Our method also provides an automated and unbiased means of evaluating therapeutic approaches and of diagnostic classification of unseen cardiac tissue. Furthermore, the wealth of quantitative data on the anatomy of microcirculation provided can serve as reference for comparisons for future studies in the field of cardiac microvascular research as well as enable modelling microcirculation at different stages of pathology. Lastly, the open-source nature of our approach makes it reusable in future studies of the cardiac microvasculature, but also in the study of other diseases and tissue types for which the microvascular structure and its changes are in focus.

## Methods

### Animals and Myocardial Infarction

All experiments were approved by the Ethical Committee for Animal Experimentation of CNIC and the Comunidad Autónoma de Madrid in accordance with the Guide for the Care and Use of Laboratory Animals. Six adult male Large-White pigs weighting 30–40 kg were anaesthetized with a ketamine/xyzaline/midazolam mixture in continuous intravenous infusion. Subsequently, acute myocardial infarction was percutaneously induced using an angioplasty balloon with 30-minute occlusion of the left anterior descending coronary artery (distal to the first diagonal branch) followed by reperfusion. The pigs were sacrificed one, three and seven days after infarction, respectively. A similar procedure with 40-minute occlusion was followed for three additional pigs that were sacrificed forty-five days following infarction. Furthermore, two more pigs under basal conditions were sacrificed to serve as the control subjects of the present study. The subjects’ structural and functional characteristics are provided in Supplementary Table S2.

### Tissue preparation and Immunofluorescence histochemistry

The porcine hearts were extracted immediately after euthanasia, cleaned with saline and cut into three thick cross-sections, at the basal, mid-ventricular (papillary muscle) and apical levels. Left ventricle tissue samples from both infarcted and remote areas were collected and used for the purposes of this study. Samples were fixed overnight with 0.4% PFA at 4 °C, washed with PBS, cryoprotected with 30% sucrose and embedded in OCT with transmural orientation kept. Thick slices of 100 *μm* were cut using Leica AM1950 automated cryostat. Immunostaining was performed in flotation using the following procedure; 2 hours permeabilization in 0.3% Triton-X 100, 0.1% Tween in PBS at RT followed by 1 hour blocking with 0.3% Triton-X 100, 4% FBS in PBS at 4 °C. For the staining of the microvasculature, the slices were incubated in blocking buffer with primary antibodies anti-VE-Cadherin (Santa Cruz) and α-SMA (Sigma) with dilution 1:100 and 1:200 respectively, overnight at 4 °C. After washing with 0.3% Triton-X 100 in PBS, incubation with secondary antibodies donkey anti-goat Alexa Fluor-568, chicken anti-mouse Alexa Fluor-647, 1:500 (Molecular probes) and Hoechst 33342, 1:10000 (Life Technologies) was performed for 2 hours in blocking buffer at RT. After washing, slices were mounted on the glass using Fluoromount G (Southern Biotech). All incubations were performed on a nutator.

VE-Cadherin stains the endothelial junctions and not the complete endothelial cell, resulting in gaps in the reconstructed blood vessels. However, it has been chosen in this work as a vasculature marker over other commonly used endothelial markers, such as Isolectin B4 or CD31 (PECAM-1), due to its specificity even in infarction. Isolectin tends to stain not only endothelial cells, but also macrophages^60^. Therefore, its use in infarcted tissue is not appropriate, especially at early time-points, when the inflammation process occurs. In our samples large contamination of macrophage staining was observed in infarct zones when Isolectin was used. A similar problem was presented in the case of CD31 marker, which is present in endothelial cell junctions but it is also expressed in other cell types, e.g. platelets, monocytes and macrophages^61^. For those reasons, the use of VE-Cadherin that is exclusively an endothelial cell marker^62^, was preferred.

A similar protocol was followed in the case of tissue used to study the co-expression of α-SMA with other markers. More precisely, 15 *μm* slices of tissue from infarcted areas from the pigs sacrificed at 7 days following MI were used. After fixation and cutting, the slices were incubated in blocking buffer with primary antibody anti-CD31 (Abcam) with dilution 1:100 and one of the following primary antibodies: (i) anti-Vimentin (Sigma) with dilution 1:100, (ii) anti-Collagen I (Santa-Cruz) with dilution 1:50, or (iii) anti-PDGFRB (eBioscience) with dilution 1:100, overnight at 4 °C. After washing with 0.3% Triton-X 100 in PBS, incubation with secondary antibodies goat anti-rabbit Alexa Fluor-488 (Life Technologies) with dilution 1:500, chicken anti-mouse Alexa Fluor-647, 1:500 (Life Technologies) and Hoechst 33342, 1:10000 (Invitrogen) was performed for 2 hours in blocking buffer at RT. After washing with 0.3% Triton-X 100 in PBS, the slices were incubated in blocking buffer with 5% mouse serum for 1 hour at RT. Subsequently, they were incubated with anti-α-SMA-Cy3 (Sigma) with dilution 1:300 for an additional hour at RT. After a final wash with 0.3% Triton-X 100 in PBS, the slices were mounted on the glass using Fluoromount G. In this case CD31 was used as a marker of the microvasculature instead of VE-Cadherin due to compatibility issues of the species of primary antibody host.

### Image acquisition

Spectral imaging was performed by means of Leica SP5 confocal microscopy using a 40 × oil immersion lens of numerical aperture 1.25. Emissions of 405 *nm*, 561 *nm*, and 633 *nm* laser lines were used to excite the Hoechst, VE-Cadherin and SMA fluorophores respectively. Z-stack slices (1024 × 1024 pixels) were acquired every 1 *μm* and by applying the deepness correction set-up provided by the microscope. The final number of slices acquired depended on particular antibody penetration and ranged between 54 and 95 *μm*. The resulting voxel size was 0.3785 *μm* × 0.3785 *μm* × 1.007 *μm*, or 0.3142 *μm* × 0.3142 *μm* × 1.007 *μm* in a few cases. In total, 126 multichannel images, 18 per tissue condition (9 per subject), were acquired and they were used for the analysis at time-points 1, 3, 7 days post MI and under basal conditions. Additionally, 36 images, 18 from infarcted (6 per subject) and 18 from remote areas (6 per subject), were acquired in the same manner and they were used for investigating microvascular characteristics and changes at 45 days following MI.

The same imaging system and procedure were also used to acquire images from tissue simultaneously labelled using anti-CD31, anti-SMA and anti-PDGFRB/anti-vimentin. However, emissions of 405 *nm*, 488 *nm*, 561 *nm*, and 633 *nm* laser lines were used to excite the Hoechst, CD31, SMA and PDGFRB or vimentin fluorophores, respectively. In the case of tissue simultaneously labelled with anti-CD31, anti-SMA and anti-collagen I, the image acquisition process followed was the same, with the difference that the Nikon A1R confocal microscopy was used.

### Multi-scale Multi-level Thresholding (MMT)

The first step towards quantifying the microvasculature and its infarction-related dynamic changes is to automatically segment the labelled structures of the VE-Cadherin, SMA and of the Hoechst channels. Towards this aim, the multi-scale multi-level thresholding algorithm^30^ is applied to every channel of the 3D confocal image.

Multi-scale multi-level thresholding has been previously described in detail^30^. In essence, grids of boxes of various sizes *e* = 1, .., *N* were applied to the original de-noised image *I*. For every grid size *e*, a candidate segmentation (*V*_*e*_) was produced by applying Otsu’s multi-level thresholding^63^ to each box comprising the grid in order to separate *I* into *M* intensity levels. Subsequently, only the two highest intensity levels were retained as part of the structure under investigation. Final segmentation was produced by using the majority rule on candidate segmentations.

In the case of the VE-Cadherin channel, the segmentation is further improved by excluding possible artefacts, i.e. objects that are smaller than 100 voxels and that do not have a nucleus.

### Fractal-based Metrics

Fractal dimension was calculated by applying the box-counting method in the 3D volume of the reconstructed microvasculature. In brief, grids of boxes of different sizes were overlaid on the microvasculature. Fractal dimension was estimated by

1 $$Fd=-\,{lim}\,\frac{{ln}(n(e))}{{ln}(e)}$$

where *n*(*e*) is the number of boxes of size *e* needed to cover the object under investigation. We subsequently identify the cut-off scales over which the microvascular structures no longer present self-similarity^64^, i.e. can not be considered as fractals. This additional step was considered necessary because real-life objects, such as the vascular patterns, might not present self-similarity over an infinite range of scales but rather over finite scales. However, no statistically significant difference was observed between the calculation of fractal dimension with and without cut-offs. This might be explained by the limited physical scales of the microvascular patterns in our dataset. Consequently, the fractal dimension presented in this manuscript is without cut-offs.

Lacunarity was calculated by using the gliding box method^36^. Boxes of various sizes *e* were glided over the 3D binary volume of the segmented microvasculature by 1 voxel. For every box size *e*, the total number of boxes of size *e* (*N*(*e*)) and the number of vascular voxels (*P*(*n*,*e*)) inside every box *n* = 1, .., *N*(*e*) were taken into account. The sum of the number of voxels belonging to the microvasculature in every box *n* = 1, ..., *N*(*e*) was calculated $({Q}_{1}={\sum }_{n=1,\mathrm{..},N(e)}P(n,e))$, as well as the sum of the square number of vascular voxels $({Q}_{2}={\sum }_{n=\mathrm{1,}\mathrm{..,}N(e)}P{(n,e)}^{2})$. Lacunarity for box size *e* was then given by

2 $$L(e)=\frac{N(e)\times {Q}_{2}}{{{Q}_{1}}^{2}}$$

In order to tackle the dependence of lacunarity on image density and to be able to compare images of various densities as the ones comprising our dataset, lacunarity was subsequently normalized using the lacunarity of the complementary image (*cL*(*e*)) by ${L}_{norm}(e)=1-\frac{1}{L(e)}-\frac{1}{cL(e)}$. Lacunarity over all scales, i.e. all box sizes, is finally defined as the mean of the normalized lacunarity of individual scales.

Succolarity was calculated according to previous study^65^. The areas where blood can flow through the microvasculature are the vascularized areas, while the non-vascularized were considered to be obstructing the flow. There are six possible directions of flow in the case of the 3D microvasculature: horizontal flow from left to right and vice versa, vertical flow from top to bottom and vice versa, and in-depth blood flow from the upper to the lower slices and vice versa. The volume of the 3D microvasculature was decomposed in six 3D images, one per flow direction. Each of those images contained only microvascular parts that would allow a continuous flow, if blood was to enter from that particular direction.

Grids of boxes of different sizes, as in the case of the box counting method, were applied on the decomposed images. The occupation percentage of vascular voxels (*O*(*n*,*e*)) inside each box *n* of size *e*, as well as an indicator of pressure (*PR*(*e*)), were calculated. *PR*(*e*) was calculated based on the coordinates of the centroid of the box along the direction under investigation. Therefore, in the cases of horizontal, vertical and in depth directions of blood flow respectively, the pressure was given by the x, y and z coordinates respectively. A normalized version of succolarity was given by dividing the pressure in the case of 100% occupation with vascular voxels for all boxes. Therefore,

3 $$S(e,d)=\frac{\sum _{n=1,\,\mathrm{...},\,N(e)}OP(n,e)\times PR(e)}{\sum _{n=1,\,\mathrm{..},\,N(e)}PR(e)}$$

Taking into account all 6 directions, the overall succolarity was approximated as

4 $$S=\frac{1}{6}\sum _{d=1,\,\mathrm{...},\,6}\sum _{e}\frac{S(d,e)}{N(e)}$$

where *N*(*e*) refers to the total number of boxes of size *e* comprising the grid.

### Minkowski Metrics

For an arbitrary object A in the d-dimensional Euclidean space ${{\mathbb{R}}}^{d}$, there exist d + 1 Minkowski Functionals (MF) *M*_*n*_(*A*) with *n* = 1, .., *d* + 1. Therefore, in 3−*D* space, there exist four MF, which are proportional to the commonly known properties volume (*M*_1_), surface area (*M*_2_), mean integral of breadth (*M*_3_) and Euler-Poincaré characteristic (*M*_4_). The MFs for the 3D microvasculature are calculated by^35^:

5 $$\begin{array}{rcl}{M}_{1}(V) & = & {n}_{c}\\ {M}_{2}(V) & = & -6{n}_{c}-2{n}_{f}\\ {M}_{3}(V) & = & 3{n}_{c}-2{n}_{f}+{n}_{e}\\ {M}_{4}(V) & = & -{n}_{c}+{n}_{f}-{n}_{e}+{n}_{v}\end{array}$$

where *n*_*c*_ is the total number of voxels that the microvasculature consists of, *n*_*f*_ is the number of open faces, *n*_*e*_ the number of edges and *n*_*v*_ the number of vertices.

In the present work, we normalize the MFs by the volume of the tissue from which the microvasculature was reconstructed to obtain density, surface area density, breadth density and Euler-Poincaré characteristic density. Normalization was performed in order to allow the comparative analysis of the MFs among different images. This would otherwise have not been possible due to the variability found in tissue volume among the images of our dataset.

### Graph-based representation and related metrics

In order to convert the microvasculature to a 3D graph that encompasses the topological information of the network, the reconstructed microvasculature is firstly skeletonized through the use of a thinning algorithm^66^. The skeleton is converted to a graph consisting of nodes that represent the branching or end nodes that could be possible sprouts (mean and standard deviations presented in Table 1) of the microvascular network and the edges (i.e. segments) that represent the skeleton parts between two consecutive nodes^67^. Subsequently, to eliminate false short spurs produced by the thinning procedure, the 3D image volume with possible areas of arterioles/venules obtained after Frangi filtering (*G*) introduced previously, is used. *G* allows us to guide a local, pruning process during which end segments (segments defined between a branching node and an ending node) are pruned based on their length and type (capillaries or arterioles/venules). More precisely, segments whose length is smaller than one-and-a-half times the diameter of the smallest capillary (4.1 *μm*), found in the LV of the pig heart according to^13^, are used in areas that the 3D *G* indicated that corresponded to capillaries. On the other hand, on those areas that correspond to arterioles/venules according to *G*, the segments are pruned when their length is smaller than one-and-a-half times the diameter of the smallest arteriole/venule of order 1 (8.96 *μm*)^13^. The pruning procedure is repeated twice to ensure that spur “Y”-shaped segments, resulting from the thinning process, are entirely pruned. The branching and end-nodes of the skeleton are extracted as nodes with more than one and only one neighbouring point respectively by convolving the 3D skeleton of the image with a 3 × 3 × 3 sized mask of ones. Bubble-like nodes resulting as an artefact of the skeletonization process are replaced by the central node. The radius of each segment is calculated using the distance of the points on the skeleton to the closest non-vascular element. Among the distances, the radius is set equal to the largest one. Every segment of microvasculature between two nodes *i*,*j* is subsequently considered a tube of constant diameter (*d*_*ij*_), length (*L*_*ij*_) and the following metrics at segment level are calculated as in^68^:

6 $$\begin{array}{rcl}Vascular\,segment\,volume\,({V}_{ij}) & = & \frac{\pi \times {d}_{ij}^{2}\times {L}_{ij}}{4}\\ Vascular\,segment\,surface\,({S}_{ij}) & = & \pi \times {d}_{ij}\times {L}_{ij}\\ Tortuosity & = & \frac{{L}_{ij}}{Euclidean\,distance\,between\,i\,and\,j}\\ Vascular\,length\,density\,({L}_{d}) & = & \frac{1}{V}\times \sum _{ij=\mathrm{1,}\,\mathrm{...,}\,N}{L}_{ij}\\ Vascular\,surface\,density & = & \frac{1}{V}\times \sum _{ij=\mathrm{1,}\,\mathrm{...,}\,N}{S}_{ij}\\ Vascular\,volume\,density\,({V}_{d}) & = & \frac{1}{V}\times \sum _{ij=\mathrm{1,}\,\mathrm{...,}\,N}{V}_{ij}\\ Diffusion\,distance\,(D) & = & \frac{1}{\sqrt{\pi \times {L}_{d}}}\end{array}$$

where *V* is the volume of the tissue and *N* is the total number of segments that form part of the microvasculature.

### Nuclei counting

The number of nuclei first had to be calculated in order to calculate the number of α-SMA^+^ and endothelial cells. For this purpose, 3D watershed transformation^69^ was applied to the segmentation of the Hoechst channel in order to separate merged nuclei. Nuclei belonging to α-SMA^+^ were considered to be the ones with simultaneous staining of α-SMA and Hoechst. Nuclei belonging to endothelial cells were considered to be then ones that were overlapping with the surface of microvessels.

It should be noted that although watershed transformation is a popular approach for nuclei segmentation, it also tends to produce errors, such as over-estimation of nuclei. However, as development of novel tools for nuclei segmentation was beyond the scope of this work, watershed performed reasonably well for our approach and we, therefore, just adapted it for the task at hand. Nonetheless it is noted that the number of nuclei in this work might, therefore, not be precise.

### Super-ellipsoids & Optimization

Super-ellipsoids are a type of 3D geometric shape that belongs to the same, more general family of geometric shapes named superquadrics^70^. Super-ellipsoids are implicitly defined by

7 $${[{(\frac{|x-Cx|}{a})}^{\frac{2}{{e}_{2}}}+{(\frac{|y-Cy|}{b})}^{\frac{2}{{e}_{2}}}]}^{\frac{{e}_{2}}{{e}_{1}}}+{(\frac{|z-Cz|}{c})}^{\frac{2}{{e}_{1}}} < =1$$

where *a*, *b*, *c* are scale factors for axis *x*, *y* and *z* respectively, *Cx*, *Cy*, *Cz* represent the centre of the super-ellipsoid, and *e*_1_, *e*_2_ control the shape of the super-ellipsoid. *e*_1_ controls the squareness along z-axis and *e*_2_ the squareness along plane x-y. Therefore, a variety of shapes can be determined by varying *e*_1_ and *e*_2_; *e*_1_ < 1, *e*_2_ < 1 result in cuboids, *e*_2_ = 1 and *e*_1_ < 1 in cylindroids, *e*_1_ = 1, *e*_2_ < 1 in pillow shapes, *e*_1_ or *e*_2_ larger than 2 in pinched shapes, *e*_1_ or *e*_2_ equal 2 in flat-beveled shapes.

Our aim was to identify a shape that the frequency distribution of distances of points inside the shape to the closest capillary (estimated distribution) best matches a given frequency distribution of maximum extravascular distances (target distribution). Toward this aim, the capillary was first approximated by a cylinder whose diameter was considered equal to the minimum extravascular distance observed. The length of the vessel was arbitrary chosen (100 *μm*). We subsequently set *Cx*, *Cy*, *Cz* as the coordinates of the centre of the vessel, *b* as the maximum extravascular distance observed, and *c* as length of the capillary, while the set of parameters *a*, *e*_1_, and *e*_2_ as the unknowns to be defined through optimization. The target of optimization was to minimize the mean square error between estimated and target distributions. The parameters were initialized so as to produce a cylinder (i.e. *e*_1_ = 1, *e*_2_ = 0.2) whose length is equal to that of the vessel (*c* = 100 *μm*) and whose diameter (i.e. *a*,*b*) is equal to that of the maximum extravascular distance. In each step of the minimization procedure, a new set of parameters and, therefore, a new shape was defined by varying the unknown parameters in predefined ranges. In particular, parameter *a* was allowed to vary in the range between the minimum and maximum extravascular distance of the target distribution. Parameters *e*_1_, and *e*_2_ were allowed to vary between [0,3] and, therefore, cover all ranges of possible shapes. The minimization procedure stopped after a minimum was achieved in the root mean square error. It should be noted that a simplified version of the target distribution (10 bins) was used. Optimization is based on *fmincon* MATLAB (Mathworks) solver and sequential quadratic programming.

### Machine learning

Multi-class classification was performed to predict the time that elapsed since the onset of MI for infarcted tissue and remote tissue (“Infarcted over time”, and “Remote over time” respectively in Table 2). In all other cases, binary classifications were performed. 9-fold cross-validation repeated 10 times was used. k-fold validation is a common model approach in machine learning that helps to avoid overfitting the training set and it is usually repeated a multitude of times to ensure different separation of folds. The idea behind k-fold cross-validation is to divide the sample in k equal folds of which k-1 are used for training and one is kept for testing.

In this work, three different classifiers were employed. K-nearest neighbours classifier (Knn) assigns an unseen object to a class, based on the labels of the k-nearest training objects that are closest (or more similar) to the object under investigation. Here, we used 1-Knn classifier, e.g. the tissue is classified according to the label of the closest training tissue. The distance metric used was euclidean distance.

Support vector machines (SVMs) are inherently binary classifiers. Standard SVMs define a hyperplane function that guaranties optimum separation, e.g. largest margin, between the (training) data of the two classes. A new object is subsequently assigned to one or the other class based on whether the predefined hyperplane function is positive or negative for that particular object. The idea of reducing the multiclass classification problem to binary classification problems has been introduced and different approaches have been developed in order to perform multi-class classification with SVMs. In this work, we adapted the one-versus-one strategy according to which SVMs are built for all possible pairwise comparisons. Each class is, therefore, compared to each other separately.

Adaboost is based on a cascade of weak classifiers (learners) used to create a strong classifier which has higher accuracy that single classifiers. During each iteration of the training procedure, a learner is added so as to weight higher misclassified examples of the previous iteration and, therefore, to redirect the focus of subsequent weak learners in those examples. The strong classifier produced by a weighted sum of the weak learners is responsible for the assignment of labels to unseen objects. In this paper, we used Knn weak classifiers.

### Statistical analysis

Statistical significance of differences in medians for pairwise comparisons was assessed by non-parametric, two-sided Wilcoxon rank sum tests. P-values were corrected for multiple testing with the Benjamini-Hochberg false discovery rate procedure which was applied to the fifteen pairwise tests performed per each quantified parameter. The number of samples per tissue category remained the same throughout this work and it equals to 18. Two sample Kolmogorov-Smirnov tests were performed to evaluate the statistical significance of differences in distributions.

### 3D Visualization

Visualization of 3D volumes (reconstructions, skeletons, distance maps) was performed with open-source softwares Paraview (http://www.paraview.org/) and ITK-SNAP (http://www.itksnap.org/).

### Code availability

The code for all modules of the image processing pipeline presented in this work is open-source, along with the supporting documentation to run the modules separately or the complete pipeline at once. The statistical analysis and the creation of the comparative plots per metric was performed with an additional module of our pipeline, which is also available. Code/libraries from previous works necessary to run the pipeline are also provided and the creators have been credited accordingly. The code was written in MATLAB (Mathworks).

## Electronic supplementary material

Supplementary Information
