# Author Correction: Deciphering microvascular changes after myocardial infarction through 3D fully automated image analysis

**DOI:** 10.1038/s41598-018-32598-6

**Published:** 2018-09-25

**Authors:** Polyxeni Gkontra, Kerri-Ann Norton, Magdalena M. Żak, Cristina Clemente, Jaume Agüero, Borja Ibáñez, Andrés Santos, Aleksander S. Popel, Alicia G. Arroyo

**Affiliations:** 1 Centro Nacional de Investigaciones Cardiovasculares Carlos III (CNIC), Madrid, 28029 Spain; 2 Department of Biomedical Engineering, School of Medicine, Johns Hopkins University, Baltimore, MD 21205 USA; 3 Centro de Investigación Biomédica en Red de Enfermedades CardioVasculares (CIBERCV), Madrid, Spain; 4 IIS-Fundación Jiménez Díaz, Madrid, Spain; 5 Biomedical Image Technologies (BIT), ETSI Telecomunicación, Universidad Politécnica de Madrid, Madrid, 28040 Spain; 6 Centro de Investigación Biomédica en Red de Bioingeniería, Biomateriales y Nanomedicina (CIBERBBN), Madrid, Spain; 7 Present Address: Division of Science, Mathematics, and Computing, Bard College, Annandale-on-Hudson, NY, 12504 USA

Correction to: *Scientific Reports* 10.1038/s41598-018-19758-4, published online 30 January 2018

The original version of this Article omitted an affiliation for Polyxeni Gkontra. The correct affiliations for Polyxeni Gkontra are listed below:

Centro Nacional de Investigaciones Cardiovasculares Carlos III (CNIC), Madrid, 28029, Spain

Biomedical Image Technologies (BIT), ETSI Telecomunicación, Universidad Politécnica de Madrid, Madrid, 28040, Spain

This has now been corrected in the HTML and PDF versions of this Article.

