# Supplementary material for: Deciphering microvascular changes after myocardial infarction through 3D fully automated image analysis
Source: Sci Rep. 2018 Jan 30;8:1854. doi: 10.1038/s41598-018-19758-4 (PMC5789835; doi:10.1038/s41598-018-19758-4)
Supplement: Supplementary file 1 — Supplementary Information [file 41598_2018_19758_MOESM1_ESM.pdf]

Supplementary Information
Deciphering microvascular changes after myocardial infarction through 3D fully automated image analysis

Polyxeni Gkontra, Kerri-Ann Norton, Magdalena M. Żak, Cristina Clemente, Jaume Agüero, Borja Ibáñez, Andrés Santos, Aleksander S. Popel and Alicia G. Arroyo*

*Correspondence and requests for materials should be addressed to A.G.A. (email: agarroyo@cnic.es).

Supplementary Figures

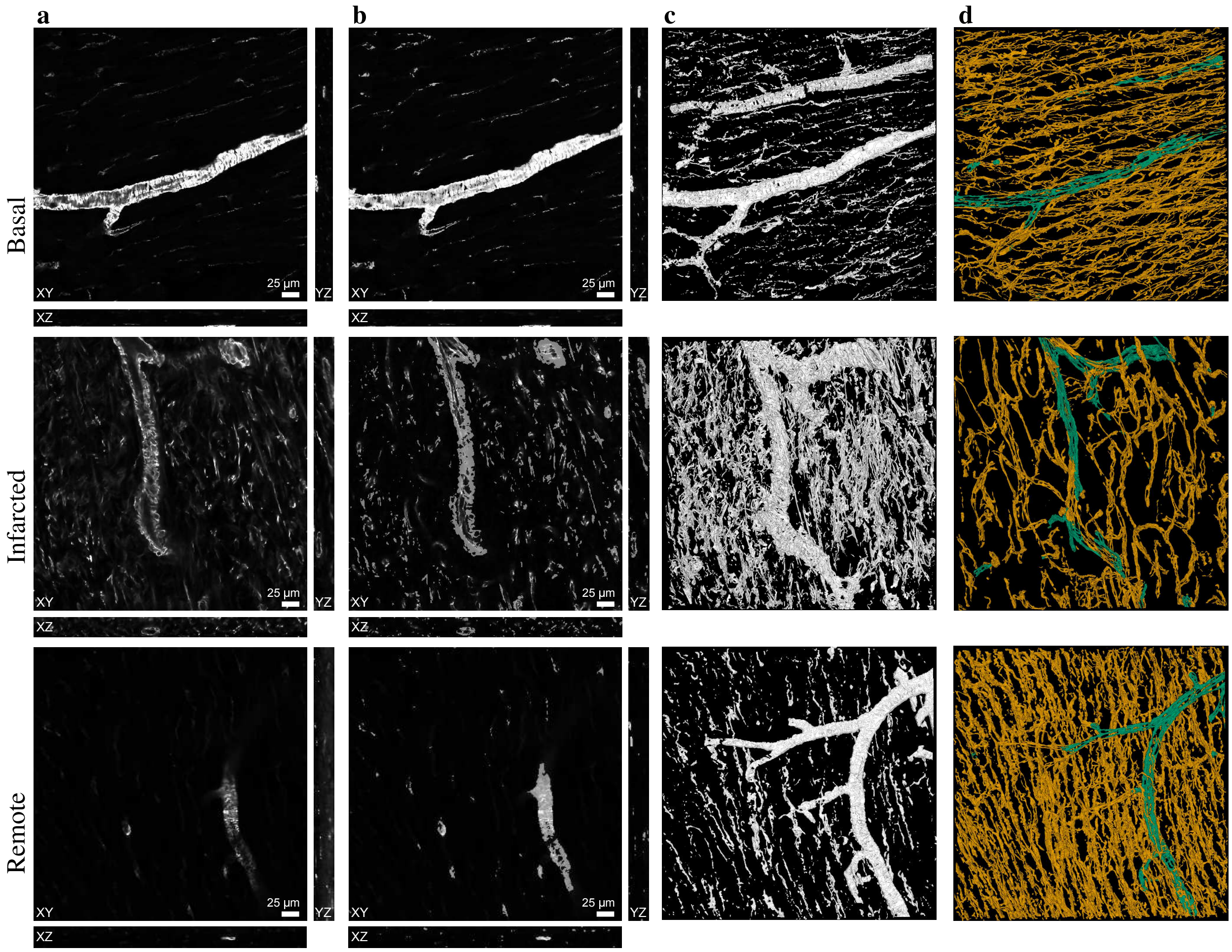

Supplementary Figure S 1. Example segmentations of the SMA^+ cells channel and the resulting 3D guidance maps for the filling procedure for a basal, an infarcted and a remote case. **(a)** Cross-sections along x-y-z of the SMA^+ cells channel. **(b)** Cross-sections along x-y-z of the SMA^+ segmentations overlaid on the original images. **(c)** SMA^+ segmentations in 3D. **(d)** 3D guidance map. Parts of the cardiac microvasculature that are recognized as capillaries are represented with orange, while those recognized as arterioles/venules are represented with green.

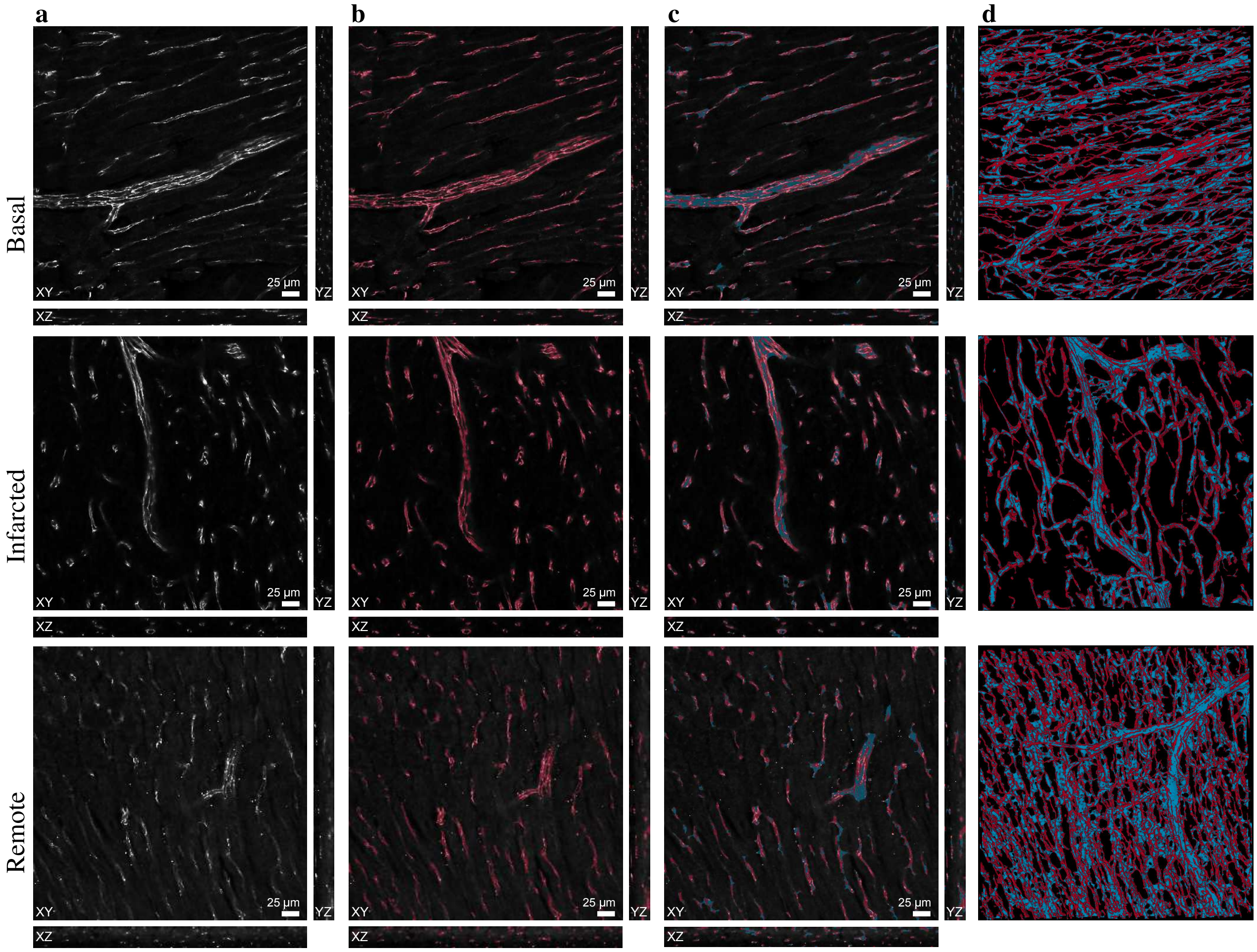

Supplementary Figure S 2. VE-Cadherin example segmentations and the corresponding reconstructed cardiac microvasculature for a basal, an infarcted and a remote case. **(a)** Cross-sections along x-y-z of the VE-Cadherin channel. **(b)** Cross-sections along x-y-z of the corresponding VE-Cadherin segmentations overlaid on the original images presented in column **a**. **(c)** Cross-sections along x-y-z of the 3D reconstructed microvasculature. **(d)** Reconstructed microvasculature in 3D. Segmented areas from the VE-Cadherin channel are shown in red, while areas reconstructed as part of the microvasculature after the application of the filling pipeline are shown in blue.

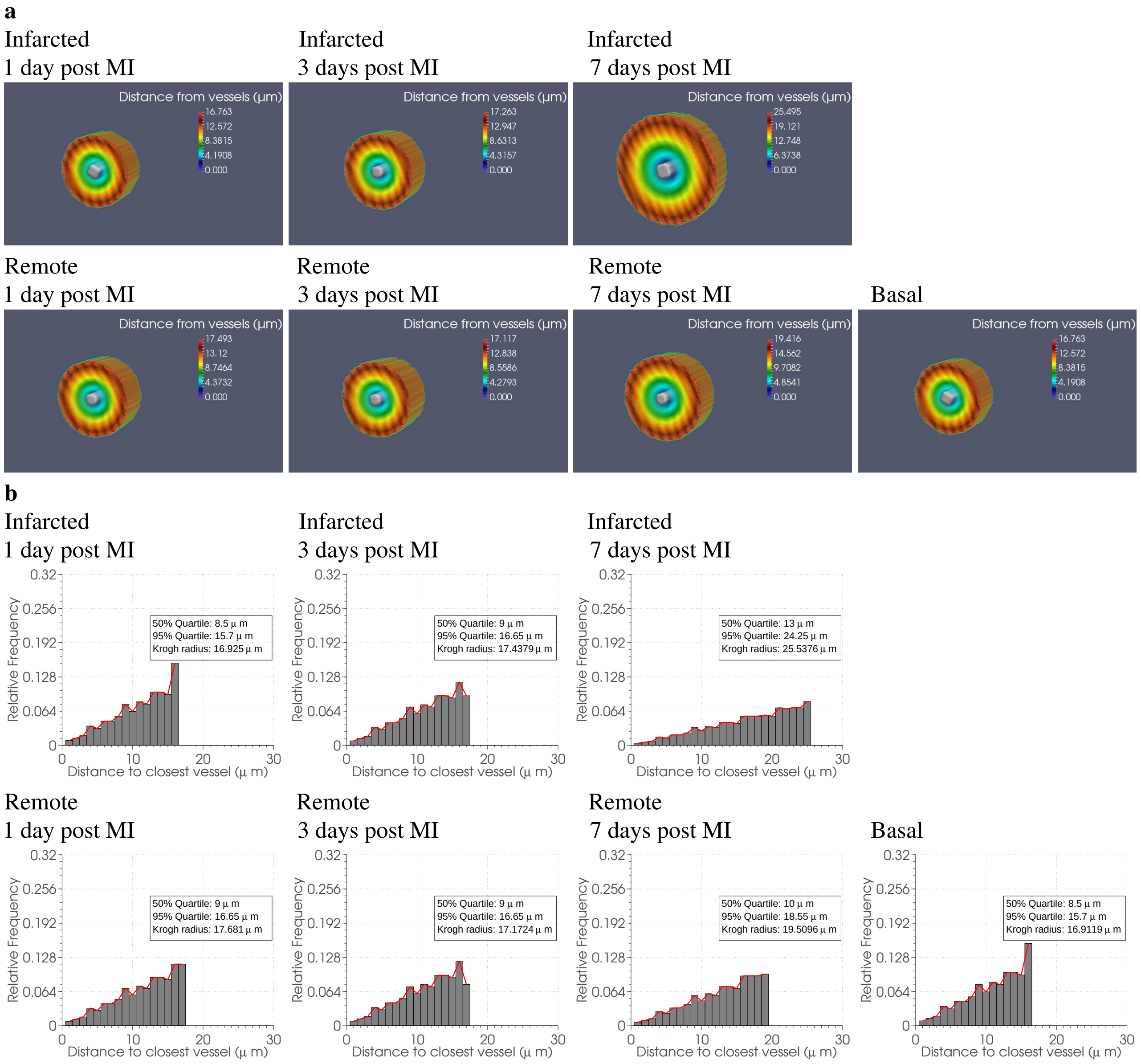

Supplementary Figure S 3. Equivalent Krogh cylinders and histograms of diffusion distances per tissue category; basal, infarcted and remote 1, 3, 7 days following MI. **(a)** Equivalent Krogh cylinders pseudo-colored according to the distance of each point to the central capillary (represented with grey in all sub-figures). The radius of the central capillary for each tissue condition was set equal to the mean vascular segment radius calculated for the particular condition. Similarly, the length of the central capillary, and thus, of the corresponding Krogh cylinder is set equal to the mean vascular segment length of the tissue condition. Finally, the radius of the Krogh cylinder is set equal to the half the intercapillary distance which was measured from capillary center to capillary center using 2D slices of the volumes of each tissue condition. **(b)** Histograms of relative frequencies of distances of the tissue points to the central capillary calculated for the equivalent Krogh cylinders of panel **a**.

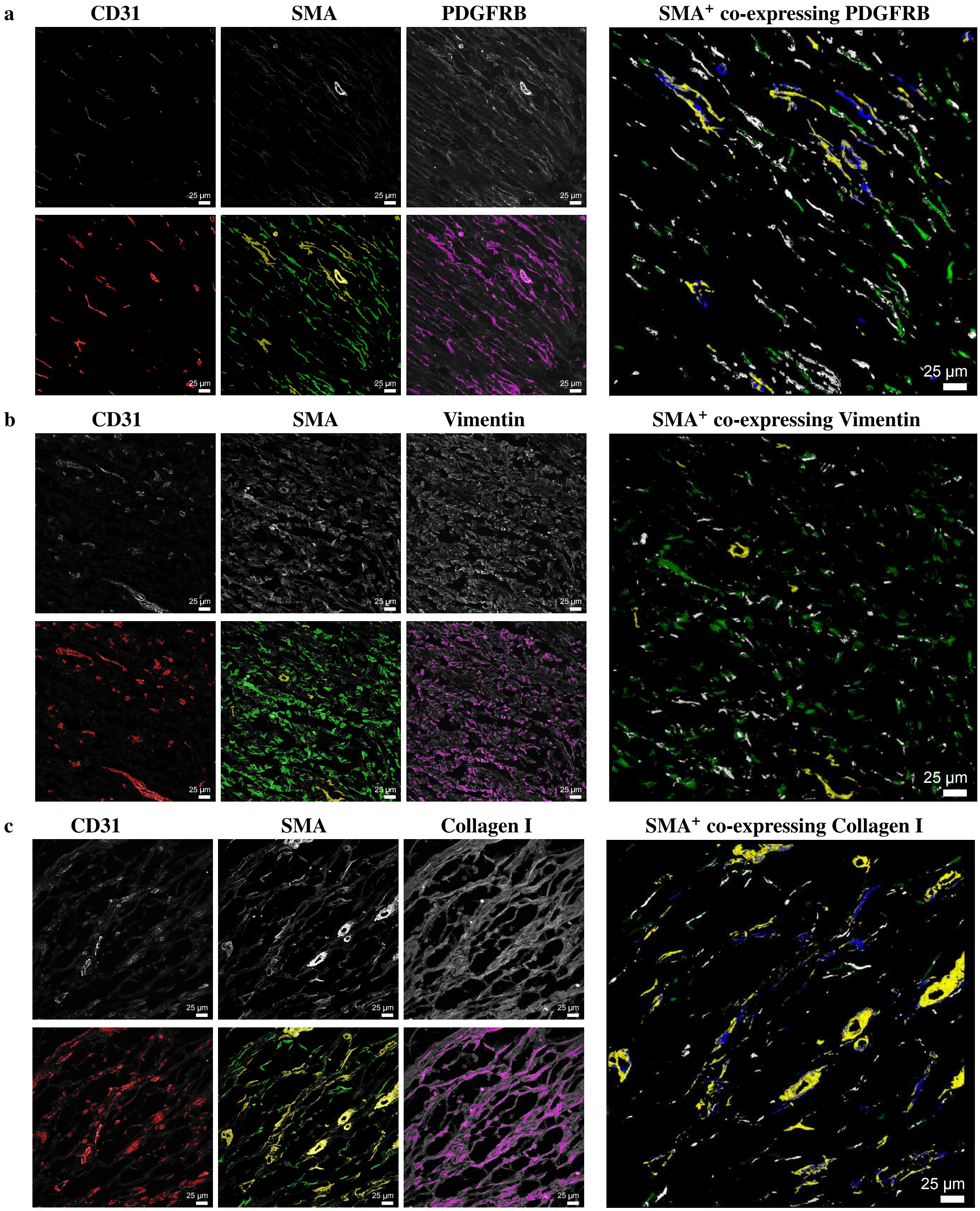

Supplementary Figure S 4. SMA$^+$ cells co-express other myofibroblast markers. **(a)** Tissue simultaneously labelled using anti-CD31, anti-SMA and anti-PDGFRB, a marker that is expressed in fibroblasts, myofibroblasts, perivascular smooth muscle cells and that increases during myocardial fibrosis. **(b)** Tissue simultaneously labelled with anti-CD31, anti-SMA and anti-vimentin, or **(c)** anti-collagen I. Both markers are expressed in fibroblasts and myofibroblasts during cardiac remodelling. In all cases, the first three columns provide the maximum intensity projections of the original channels (upper row of the panels) and of the resulting segmentations of the channels (lower rows of the panels). In the case of SMA$^+$ cell segmentation, yellow represents areas automatically recognized as SMA$^+$ perivascular regions, while green the non-vessel touching SMA$^+$ myofibroblast region. To the right, 3D reconstructions showing non-perivascular SMA+ myofibroblast areas that are positive for the other marker in grey and non-perivascular SMA+ myofibroblasts negative for the other marker in green. Perivascular SMA$^+$ area is highlighted in yellow with double-positive regions for other markers in blue.

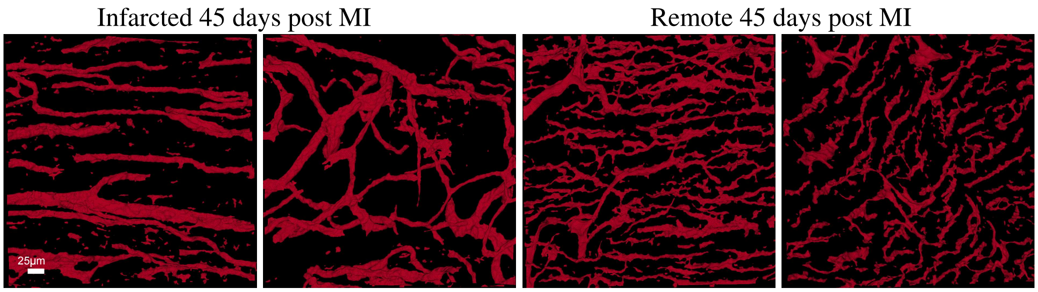

Supplementary Figure S 5. Representative 3D reconstructions of the cardiac microvasculature from tissues at 45 days post MI. The first two volumes correspond to tissues from infarcted areas, while the third and fourth volumes to tissues from remote ones.

Supplementary Tables

Supplementary Table S 1. Mean $\pm$ standard deviation of all parameters for tissue from infarcted and remote areas at 45 days following MI and their change (increase or decrease) with respect to infarcted and remote areas at earlier time-points after MI and under basal conditions. I1MI, I3MI, I7MI, and I45MI refer to infarcted areas at 1 day, 3, 7 and 45 days post MI respectively. Similarly, R1MI, R3MI, R7MI and R45MI refer to the corresponding remote areas. Up-arrows and down-arrows indicate increase and decrease respectively of the parameter value at 45 days post MI in infarcted (columns 2 to 5) or remote areas (columns 7 to 10) compared with the tissue category under investigation. Red (for increase) and blue (for decrease) colors are used to denote that the difference is statistically significant. *, ** and *** represent p-value$<$ 0.05, 0.01 and 0.001 respectively. The p-values were calculated by means of Wilcoxon rank-sum tests and corrected with the Benjamini-Hochberg procedure for multiple testing. The number of samples compared is 18 per tissue condition.

	I45MI	I45MI vs I1MI	I45MI vs I3MI	I45MI vs I7MI	I45MI vs Basal	R45MI	R45MI vs R1MI	R45MI vs R3MI	R45MI vs R7MI	R45MI vs Basal
Fractal-Based Metrics										
Fractal Dimension	2.12±0.06	$\downarrow^{***}$	$\downarrow^{***}$	$\downarrow$	$\downarrow^{***}$	2.17±0.04	$\downarrow^{***}$	$\downarrow^{***}$	$\downarrow^{**}$	$\downarrow^{**}$
Lacunarity ($\times 10^{-2}$)	89.17±4.05	$\uparrow^{***}$	$\uparrow^{***}$	$\uparrow^{***}$	$\uparrow^{***}$	85.17±5.52	$\uparrow^{***}$	$\uparrow^{***}$	$\uparrow^{***}$	$\uparrow^{***}$
Succolarity ($\times 10^{-2}$)	0.14±0.08	$\downarrow^{***}$	$\downarrow^{**}$	$\downarrow$	$\downarrow^{***}$	0.24±0.16	$\downarrow$	$\downarrow$	$\downarrow$	$\downarrow$
Minkowski-Based Metrics										
Vascular Volume Density (%)	4.88±1.24	$\downarrow^{***}$	$\downarrow^{***}$	$\downarrow$	$\downarrow^{***}$	7.03±1.11	$\downarrow$	$\uparrow^{*}$	$\uparrow$	$\uparrow$
Surface Area Density ($\times 10^{-3}$) ($\mu m^2/\mu m^3$)	21.48±5.33	$\downarrow^{***}$	$\downarrow^{***}$	$\downarrow^{**}$	$\downarrow^{***}$	39.94±5.92	$\downarrow^{**}$	$\downarrow^{**}$	$\downarrow$	$\downarrow^{**}$
Breadth Density ($\times 10^{-3}$) ($\mu m/\mu m^3$)	0.5±0.13	$\downarrow^{***}$	$\downarrow^{***}$	$\downarrow^{**}$	$\downarrow^{***}$	1.05±0.15	$\downarrow^{***}$	$\downarrow^{***}$	$\downarrow^{*}$	$\downarrow^{***}$
Euler Characteristic Density ($1/\mu m^3$)	0.97±0.99	$\uparrow^{***}$	$\uparrow^{***}$	$\uparrow$	$\uparrow^{***}$	-1.98±0.91	$\uparrow^{**}$	$\uparrow^{**}$	$\uparrow^{*}$	$\uparrow^{***}$
Capillary Volume Density (%)	4.45±1.22	$\downarrow^{***}$	$\downarrow^{**}$	$\uparrow$	$\downarrow^{***}$	6.01±1.33	$\downarrow$	$\uparrow$	$\uparrow$	$\downarrow$
Capillary Surface Area Density ($\times 10^{-3}$) ($\mu m^2/\mu m^3$)	20.78±5.36	$\downarrow^{***}$	$\downarrow^{***}$	$\downarrow^{**}$	$\downarrow^{***}$	38.23±6.44	$\downarrow^{**}$	$\downarrow^{**}$	$\downarrow$	$\downarrow^{**}$
Graph-Based Metrics										
Vascular length density ($\times 10^{-3}$)($\mu m/\mu m^3$)	1.18±0.39	$\downarrow^{***}$	$\downarrow^{***}$	$\downarrow^{**}$	$\downarrow^{***}$	2.51±0.62	$\downarrow^{**}$	$\downarrow^{***}$	$\downarrow$	$\downarrow^{**}$
Vascular surface density ($\times 10^{-3}$)($\mu m^2/\mu m^3$)	21.09±6.38	$\downarrow^{***}$	$\downarrow^{***}$	$\downarrow^{*}$	$\downarrow^{***}$	35.68±8.84	$\downarrow$	$\downarrow$	$\downarrow$	$\downarrow$
Vascular volume density ($\times 10^{-2}$)($\mu m^3/\mu m^3$)	3.56±1.02	$\downarrow^{**}$	$\downarrow$	$\uparrow$	$\downarrow$	4.61±1.24	$\uparrow$	$\uparrow^{*}$	$\uparrow$	$\uparrow$
Vascular segment radius (μm)	3.89±0.29	$\uparrow^{***}$	$\uparrow^{***}$	$\uparrow^{***}$	$\uparrow^{***}$	3.28±0.21	$\uparrow^{***}$	$\uparrow^{***}$	$\uparrow^{***}$	$\uparrow^{***}$
Vascular segment length (μm)	14.38±3.24	$\downarrow$	$\downarrow^{*}$	$\downarrow$	$\downarrow^{*}$	16.38±1.88	$\downarrow$	$\downarrow^{*}$	$\downarrow$	$\downarrow^{*}$
Vascular segment surface (μm^2)	261.44±64.16	$\uparrow^{*}$	$\uparrow^{**}$	$\uparrow$	$\uparrow$	232.39±25.64	$\uparrow$	$\uparrow$	$\uparrow^{*}$	$\uparrow$
Vascular segment volume (μm^3)	444.73±119.39	$\uparrow^{***}$	$\uparrow^{***}$	$\uparrow^{***}$	$\uparrow^{***}$	299.56±40.88	$\uparrow^{*}$	$\uparrow^{***}$	$\uparrow^{***}$	$\uparrow^{**}$
Vascular segments ($\times 10^5$)[a]	0.88±0.41	$\downarrow^{***}$	$\downarrow^{***}$	$\downarrow$	$\downarrow^{***}$	1.56±0.46	$\downarrow$	$\downarrow$	$\downarrow$	$\downarrow$
Vessels of diameter $<=$ 6.9 (μm) (%)	67.49±7.19	$\downarrow^{***}$	$\downarrow^{***}$	$\downarrow^{***}$	$\downarrow^{***}$	86.02±4.61	$\downarrow^{**}$	$\downarrow^{***}$	$\downarrow^{***}$	$\downarrow^{***}$
Vessels of diameter between 6.9 and 8.2 (μm) (%)	12.77±3.2	$\uparrow^{***}$	$\uparrow^{***}$	$\uparrow^{***}$	$\uparrow^{***}$	7.09±1.9	$\uparrow^{***}$	$\uparrow^{***}$	$\uparrow^{***}$	$\uparrow^{***}$
Vessels of diameter $>$ 8.2 (μm) (%)	19.75±7.09	$\uparrow^{***}$	$\uparrow^{***}$	$\uparrow^{***}$	$\uparrow^{***}$	6.9±3.05	$\uparrow^{*}$	$\uparrow^{***}$	$\uparrow^{**}$	$\uparrow^{***}$
SMA^+ related metrics										
Damage Index	0.13±0.06	$\uparrow^{***}$	$\uparrow^{***}$	$\downarrow$	$\uparrow^{***}$	0.01±0.01	$\downarrow$	$\downarrow$	$\downarrow$	$\downarrow$
Vessels covered with SMA (%)	54.87±13.87	$\downarrow$	$\downarrow^{**}$	$\uparrow$	$\downarrow^{***}$	81.53±8.04	$\uparrow$	$\uparrow$	$\uparrow$	$\uparrow$
SMA^+layer thickness (μm)	9.44±4.39	$\uparrow^{***}$	$\uparrow^{***}$	$\downarrow$	$\uparrow^{***}$	2.21±0.51	$\downarrow$	$\downarrow^{*}$	$\downarrow^{*}$	$\downarrow$
Myofibroblasts ($\times 10^4$)[a]	1.51±0.88	$\uparrow^{*}$	$\uparrow$	$\downarrow^{*}$	$\uparrow$	0.38±0.18	$\downarrow^{*}$	$\downarrow^{***}$	$\downarrow^{*}$	$\downarrow^{***}$
SMA^+ perivascular cells[b]	52.78±31.2	$\uparrow^{**}$	$\uparrow^{**}$	$\uparrow$	$\uparrow^{*}$	29.89±8.79	$\uparrow$	$\uparrow^{*}$	$\uparrow$	$\downarrow$
Efficiency in oxygen diffusion										
Maximal Extravascular Distance (μm)	56.32±11.2	$\uparrow^{***}$	$\uparrow^{***}$	$\uparrow^{*}$	$\uparrow^{***}$	30.91±3.43	$\uparrow$	$\uparrow^{*}$	$\uparrow$	$\uparrow^{**}$
Median Extravascular Distance (μm)	29.82±5.9	$\uparrow^{***}$	$\uparrow^{***}$	$\uparrow^{*}$	$\uparrow^{***}$	16.45±1.8	$\uparrow$	$\uparrow^{*}$	$\uparrow$	$\uparrow^{**}$
Capillary Density [c]	482.47±190.43	$\downarrow^{***}$	$\downarrow^{**}$	$\uparrow$	$\downarrow^{*}$	770.16±450.86	$\downarrow$	$\downarrow$	$\downarrow$	$\downarrow$
Intercapillary Distance (μm)	32.19±8.52	$\uparrow^{***}$	$\uparrow^{***}$	$\uparrow^{*}$	$\uparrow^{***}$	19.54±2.28	$\uparrow^{*}$	$\uparrow^{**}$	$\uparrow$	$\uparrow^{**}$
Diffusion Distance (μm)	17±2.43	$\uparrow^{***}$	$\uparrow^{***}$	$\uparrow^{**}$	$\uparrow^{***}$	11.51±1.47	$\uparrow^{**}$	$\uparrow^{***}$	$\uparrow$	$\uparrow^{**}$

[a] Number per mm^3 of tissue
[b] Number per mm vascular length
[c] Number per mm^2 of tissue

Supplementary Table S 2. Characteristics of the subjects used for the analysis.

Subject	1	2	3	4	5	6	7	8	9	10	11
Time after infarction	Basal	Basal	24 hours	24 hours	3 days	3 days	7 days	7 days	45 days	45 days	45 days
Body weight (Kg)	34	30.50	34	32.50	40	34	47.50	39.50	56.5	53	57
Left ventricle (LV) mass (g)	55.83	60.50	49.95	57.84	71.18	56.65	88.90	60.60	70.89	60.59	56.88
LV end diastolic volume (ml)	82.63	88.00	95.35	117.3	100.08	96.41	117.51	138.51	128.42	134.9	130.47
LV end systolic volume (ml)	43.20	42.70	53	77.87	44.04	59.61	62.22	87.15	83.85	93.9	87.37
Ejection Fraction (%)	47.74	51.40	44.41	33.61	56	38.17	47.05	37.08	34.71	30.39	33.04
Infarct Size LGE (%)	0	0	29	19.17	16.61	31.01	25.38	41.59	33.37	35.41	29.01
Hematocrit (%)	30.2	32.6	31.1	21.8	29.2	28	30.4	29	33.1	32.3	30.4
Blood Flow LW / R [a] ($ml/min/100g$)	121.9	86.9	135.5	142.5	N/A [b]	103.50	114.1	159.40	175.5	139.1	134.8
Blood Flow I [c] ($ml/min/100g$)	-	-	59.7	104.6	N/A [b]	107.00	58.9	50.90	29.9	79.3	88.1

[a] LW / R refer to measurements taken from the LV lateral wall (LW) for the control subjects one and two, and to measurements taken from the remote areas (R) for subjects that have suffered infarction (subjects three to eleven).

[b] N/A: Information not available due to missing MRI sequence

[c] I refers to measurements taken from the infarcted areas (I) for subjects that have suffered infarction.
